# Changes in pontine and preBötzinger/Bötzinger complex neuronal activity during remifentanil-induced respiratory depression in decerebrate dogs

**DOI:** 10.3389/fphys.2023.1156076

**Published:** 2023-06-08

**Authors:** Barbara Palkovic, Sanda Mustapic, Ivana Saric, Eckehard A. E. Stuth, Astrid G. Stucke, Edward J. Zuperku

**Affiliations:** ^1^ Department of Anesthesiology, Medical College of Wisconsin, Milwaukee, WI, United States; ^2^ Faculty of Medicine, University of Osijek, Osijek, Croatia; ^3^ University Hospital Dubrava, Zagreb, Croatia; ^4^ University Hospital Split, Split, Croatia; ^5^ Children’s Wisconsin, Milwaukee, WI, United States; ^6^ Clement J Zablocki Department of Veterans Affairs Medical Center, Milwaukee, WI, United States

**Keywords:** opioid-induced respiratory depression, respiratory control, preBötzinger complex, pontine respiratory group, respiratory pattern generator

## Abstract

**Introduction:** *In vivo* studies using selective, localized opioid antagonist injections or localized opioid receptor deletion have identified that systemic opioids dose-dependently depress respiratory output through effects in multiple respiratory-related brainstem areas.

**Methods:** With approval of the subcommittee on animal studies of the Zablocki VA Medical Center, experiments were performed in 53 decerebrate, vagotomized, mechanically ventilated dogs of either sex during isocapnic hyperoxia. We performed single neuron recordings in the Pontine Respiratory Group (PRG, *n* = 432) and preBötzinger/Bötzinger complex region (preBötC/BötC, *n* = 213) before and during intravenous remifentanil infusion (0.1–1 mcg/kg/min) and then until complete recovery of phrenic nerve activity. A generalized linear mixed model was used to determine changes in Fn with remifentanil and the statistical association between remifentanil-induced changes in Fn and changes in inspiratory and expiratory duration and peak phrenic activity. Analysis was controlled via random effects for animal, run, and neuron type.

**Results:** Remifentanil decreased Fn in most neuron subtypes in the preBötC/BötC as well as in inspiratory (I), inspiratory-expiratory, expiratory (E) decrementing and non-respiratory modulated neurons in the PRG. The decrease in PRG inspiratory and non-respiratory modulated neuronal activity was associated with an increase in inspiratory duration. In the preBötC, the decrease in I-decrementing neuron activity was associated with an increase in expiratory and of E-decrementing activity with an increase in inspiratory duration. In contrast, decreased activity of I-augmenting neurons was associated with a decrease in inspiratory duration.

**Discussion:** While statistical associations do not necessarily imply a causal relationship, our data suggest mechanisms for the opioid-induced increase in expiratory duration in the PRG and preBötC/BötC and how inspiratory failure at high opioid doses may result from a decrease in activity and decrease in slope of the pre-inspiratory ramp-like activity in preBötC/BötC pre-inspiratory neurons combined with a depression of preBötC/BötC I-augmenting neurons. Additional studies must clarify whether the observed changes in neuronal activity are due to direct neuronal inhibition or decreased excitatory inputs.

## Introduction

The potential for morbidity and mortality when opioids are used for perioperative pain, chronic pain, or in a substance use disorder has led to intense investigation of the mechanisms of opioid-induced respiratory depression. There is agreement that clinically relevant opioid doses affect respiratory control at multiple levels (Palkovic et al., 2020). In awake human volunteers, analgesic doses of morphine depressed the peripheral component of the hypoxic ventilatory response and hypercapnic ventilatory response in women and the central component of the hypercapnic response (apneic threshold) in men (Dahan et al., 1998; Sarton et al., 1999). This was in line with results from phenobarbital-anesthetized cats where mu- and delta opioid receptor agonists decreased carotid body discharge (Kirby and McQueen, 1986). Animal studies suggest additional inhibition of hypoxic inputs at the level of the nucleus of the solitary tract (Zhang et al., 2011).

Nuclear injections of opioid antagonists during intravenous remifentanil infusion (Mustapic et al., 2010; Prkic et al., 2012; Stucke et al., 2015; Miller et al., 2017; Palkovic et al., 2021; Palkovic et al., 2022) or systemic opioids in mice with localized mu-opioid receptor knockout (Bachmutsky et al., 2020; Varga et al., 2020), resp., demonstrated that the Pontine Respiratory Group (PRG), consisting of the parabrachial and Kölliker-Fuse nuclei, the preBötzinger Complex (preBötC), and likely the caudal medullary raphe were affected by systemically administered opioids. The studies highlighted that the relevance of individual areas for opioid-induced respiratory depression was dose-dependent, that respiratory rhythm generation and tidal volume were affected differentially, and that at least very high opioid concentrations could still depress respiratory rate and tidal volume even after opioid reversal with naloxone microinjections in the PRG, preBötC, and caudal medullary raphe (Palkovic et al., 2021; Palkovic et al., 2022).

While nuclear opioid agonist and antagonist injections can identify the brainstem areas affected by systemic opioids, they do not allow conclusions regarding the neuronal mechanisms of opioid-induced respiratory depression. Multiple studies investigated systemic opioid effects on single respiratory neurons in the medullary ventral respiratory group *in vivo*. Intracellular recordings in propriobulbar and bulbospinal neurons showed dose-dependent effects where only high opioid doses caused direct depression of medullary neurons (Haji et al., 2003; Lalley, 2006), suggesting that at lower doses a decrease in neuronal discharge frequency and respiratory rate was due to a decrease in excitatory inputs, e.g., from the PRG. Apnea-inducing doses of fentanyl completely depressed activity of inspiratory neurons in the Kölliker-Fuse Nucleus in the *in-situ* rat preparation while activity of expiratory neurons and in particular expiratory plateau neurons was little depressed (Saunders et al., 2022). These types of single-cell recordings are extremely difficult to perform, and the resulting numbers are not sufficient to determine statistical associations between changes in neuronal discharge activity and concomitant changes in respiratory parameters. The present study used extracellular multichannel recordings to determine the effect of intravenous infusion of the mu-opioid receptor agonist remifentanil on neuronal discharge frequency in the PRG and preBötC and Bötzinger Complex (preBötC/BötC) areas. Since there is no clear anatomic separation between the preBötC and BötC, we recorded neurons in both areas. Our primary goal was to address three questions that were based on previous studies in the decerebrate dog and rabbit models (Mustapic et al., 2010; Prkic et al., 2012; Zuperku et al., 2019; Palkovic et al., 2021; Palkovic et al., 2022):

1 - Is the marked increase in expiratory phase duration (TE) that is observed with intravenous remifentanil associated with a depression of preBötC/BötC pre-I neurons? Pre-inspiratory (pre-I) neuron activity in the preBötC/BötC has been proposed to be an important contributor to inspiratory on-switch and thus a determinant of expiratory duration (Onimaru and Homma, 2006; Zuperku et al., 2019).
2 - Is there a neuronal mechanism that limits the increase in inspiratory duration (TI) with opioid exposure? Systemic opioids increase TI and TE, however, higher doses mainly increase TE (Prkic et al., 2012; Palkovic et al., 2021). DAMGO injection into the PRG increases TI and TE, and naloxone injection into the PRG during intravenous remifentanil decreases TI and TE in the dog and rabbit (Prkic et al., 2012; Miller et al., 2017; Palkovic et al., 2021). Mu-opioid receptor agonist injections directly into the preBötC/BötC caused a decrease in TI and TE, relative to baseline, in *in vivo* dogs (Mustapic et al., 2010) and rabbits (Stucke et al., 2015). Microdialysis of mu-agonist into the preBötC of awake and asleep goats decreased TI and increased TE (Langer et al., 2017), and decreased TI but did not change TE in rats (Montandon et al., 2011). Mu-agonist injection into the bilateral Kölliker-Fuse nucleus increased TI and TE in rats (Levitt et al., 2015), and opioid antagonist injection decreased TI and TE, which were increased from systemic fentanyl (Saunders and Levitt, 2020) in rabbits, naloxone injection into the preBötC/BötC further increased rather than reversed the increase in inspiratory duration from apneic doses of remifentanil (Palkovic et al., 2021).
3 - Is opioid-induced depression of peak phrenic activity (PPA) associated with decreased activity in PRG or preBötC/BötC I neurons? Naloxone injection into the PRG partially reversed the remifentanil-induced depression of PPA in decerebrate rabbits (Palkovic et al., 2021; Palkovic et al., 2022) but not in dogs (Prkic et al., 2012).

## Methods

### Surgical preparation

The research was approved by the subcommittee on animal studies of the Zablocki Department of Veterans Affairs (VA) Medical Center in accordance with provisions of the Animal Welfare Act, the Guide for the Care and Use of Laboratory Animals, and VA policy. Experiments were performed on 53 beagle or mongrel dogs of either sex, weighing 5–15 kg. General anesthesia was induced by mask with 5% isoflurane, animals were endotracheally intubated, and anesthesia was maintained with isoflurane at 1.5%–2.5%. Animals were ventilated with an air-oxygen mixture and maintained in hyperoxic hypercapnia (FiO_2_ > 0.6, end-tidal CO_2_ 40–50 mmHg) using an anesthesia machine (Ohmeda CD, GE, Datex Ohmeda, United States). Inspiratory oxygen fraction and end-tidal concentration of carbon dioxide and isoflurane were continuously displayed with an infrared analyzer (POET II, Criticare Systems, United States). Anesthetic depth was increased immediately for signs of inadequate anesthesia such as salivation, lacrimation, and increases in blood pressure and heart rate. The femoral artery was cannulated for blood pressure recording and blood gas sampling, and the femoral vein for maintenance fluid infusion and drug administration. Esophageal temperature was maintained at 38.5 ± 1°C with a warming pad. Mean arterial pressure was maintained above 80 mmHg with adjustment of intravenous fluids or, if required, phenylephrine infusion (0.5–5 mcg/kg/min). Animals were positioned in a stereotaxic device (model 1530, David Kopf Instruments, Tujunga, CA) with the head ventrally flexed (30°). A bilateral pneumothorax was performed to minimize brainstem movement and input from chest wall mechanoreceptors. Animals were decerebrated by midcollicular transection (Tonkovic-Capin et al., 1998), and isoflurane was discontinued. For the preBötC/BötC study, the dorsal surface of the medulla oblongata was exposed by an occipital craniotomy and removal of the caudal end of the cerebellum. For the PRG study, complete access to the dorsal surface of the brainstem was achieved by removal of the external sagittal and nuchal bone crest and cerebellectomy. After bilateral neck dissections, phrenic nerve activity was recorded from the desheathed right C5 rootlet. Bilateral vagotomy was performed to achieve peripheral deafferentation. Continuous neuromuscular blockade was achieved with pancuronium (0.1 mg/kg/h) to reduce motion artefacts during neuronal recording. At the end of the experiment, the animals were euthanized with intravenous potassium chloride.

### Neuronal recordings and measured variables

The phrenic neurogram was obtained from the moving-time average (100 ms) of the amplified, rectified phrenic nerve activity and was used to provide timing signals at the beginning and end of the inspiratory phase to determine inspiratory duration (TI), expiratory duration (TE), and peak phrenic activity (PPA). Respiratory rate was calculated as 60/(TI + TE). Phrenic neurogram, respiratory rate, arterial blood pressure, and end-tidal carbon dioxide concentration were also continuously recorded on a computerized chart recorder (Powerlab/16SP; ADInstruments, Australia). Respiratory neuronal activity was recorded either from the ventral respiratory column or from the pontine respiratory group. Early in the preBötC/BötC study, single neurons were recorded extracellularly using a custom-made multibarrel electrode with 7 μm carbon filament. All later recordings used a NeuroNexus 16-electrode probe (A1x16-10 mm-100-177-A16; www.neuronexus.com). The 16 electrodes were linearly arranged with an inter-electrode spacing of 100 μm. The probe output connected directly with a dual inline socket equipped with 16 miniature preamplifiers (4x gain; Tucker-Davis model RA16AC4; www.tdt.com). The preamplified signals were further amplified (×3000) and bandpass filtered (0.3–3 kHz; CWE 16-channel system 2000; www.cwe-inc.com). The signals were digitized at 20 kHz/channel with 16-bit resolution with a Cambridge Electronic Design CED 1401 mk II system and displayed and analyzed using Spike2 software (version 7.16). Spike2 was used for spike sorting (template matching and principal component analysis) and generating interspike interval histograms, auto-correlograms, cycle-triggered histograms (bin 50 ms), and averages of PPA. Cycle-triggered histo grams (>20 cycles) of neuronal discharge activity were generated before, during and after remifentanil infusion. Data were exported from Spike2 into SigmaPlot 11 (Systat Software, San Jose, CA) for data reduction, plotting, and statistical analysis.

**FIGURE 1:**
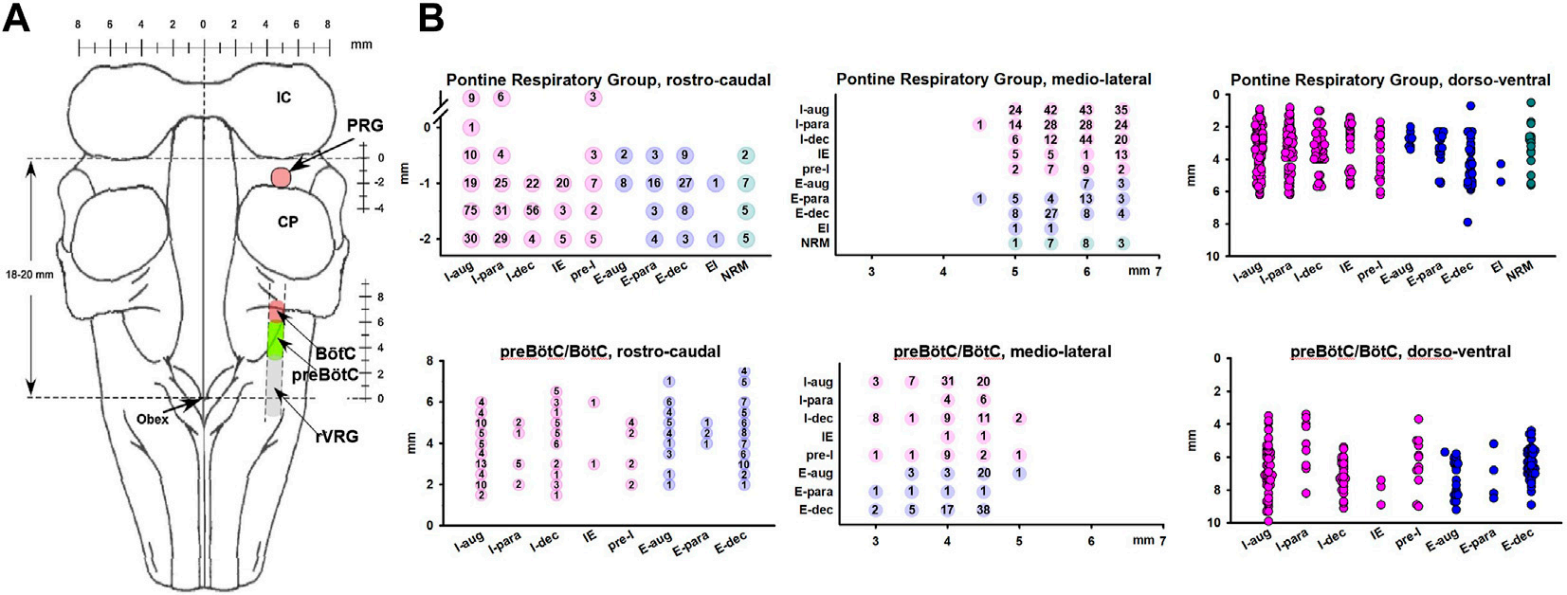
**(A)** Dorsal view of the brainstem showing the neuron recording sites. **(B)** Anatomical location for each neuron, separated by subtypes and location in the Pontine Respiratory Group (upper) and preBötzinger/Bötzinger Complex area (preBötC/BötC, lower). Coordinates reflect the grid-wise exploration of the brainstem with 0.5 mm steps rostro-caudal and medio-lateral. The number of neurons recorded at each coordinate is provided. Locations in the dorso-ventral dimension reflect the layout of the multichannel electrode, i.e., neurons could be recorded in 100-μm intervals. Neurons recorded at the same coordinate are depicted individually and staggered for clarity. The preBötC/BötC likely included neurons of the rostral ventral respiratory group (rVRG). In rostro-caudal dimensions, for the Pontine Respiratory Group, 0 mm indicates the caudal end of the inferior collicle (IC) and for the preBötC, 0 mm indicates obex. Neurons were categorized by their main discharge phase as inspiratory (I, pink), expiratory (E, blue), or non-respiratory modulated (NRM, green). Discharge patterns were described as augmenting (aug), parabolic (para), decrementing (dec), pre-inspiratory (pre-I), inspiratory-expiratory (IE), and expiratory-inspiratory (EI). CP, cerebellar peduncle.

Neuronal recordings in the preBötC/BötC study were performed in the area previously identified through stereotaxic coordinates, the presence of a mixture of respiratory neuron subtypes, the tachypneic response to D-homocysteic acid (DLH) microinjections (20 mM, 30–40 nL), and posthoc histological confirmation (Krolo et al., 2005; Mustapic et al., 2010). The preBötC/BötC was located approximately 4–7 mm rostral from obex, 4–5 mm lateral to the midline, and 6–8 mm ventral to the dorsal surface. To minimize tissue disruption from multiple DLH injections, the present study used multiple sets of recordings at 3–8 mm rostral from obex with probe insertions spaced in a grid-wise fashion 0.5–1 mm apart rostro-caudally as well as medio-laterally, accepting that the recorded neurons may include some bulbospinal neurons at the caudal end and neurons belonging to the BC and retrofacial regions at the rostral end of the range. Remifentanil infusion protocols were performed with the probe in areas where a mix of neuron types was found, which was characteristic of the area where DLH injections elicited tachypnea (Krolo et al., 2005; Mustapic et al., 2010). Stereotaxic coordinates of the probe tip, the location of each of the 16 electrodes and the tilt angle of the medulla for each dog were used to assign a location to each recorded neuron (Figure 1).

Neuronal recordings in the PRG study were performed in the area 0–3 mm caudal to the caudal pole of the inferior colliculus and 4–7 mm lateral from midline where respiratory modulated neurons were recorded at a depth of 2–6 mm. In a prior study, DAMGO injection into this area had caused bradypnea, and naloxone injection had reversed the bradypneic effect of intravenous remifentanil (Prkic et al., 2012). Posthoc histology had located the area in the medial parabrachial nucleus (Prkic et al., 2012), however, since the current study did not perform additional confirmatory histology, we refer to the area as part of the pontine respiratory group (PRG).

### Neuron classification

**FIGURE 2:**
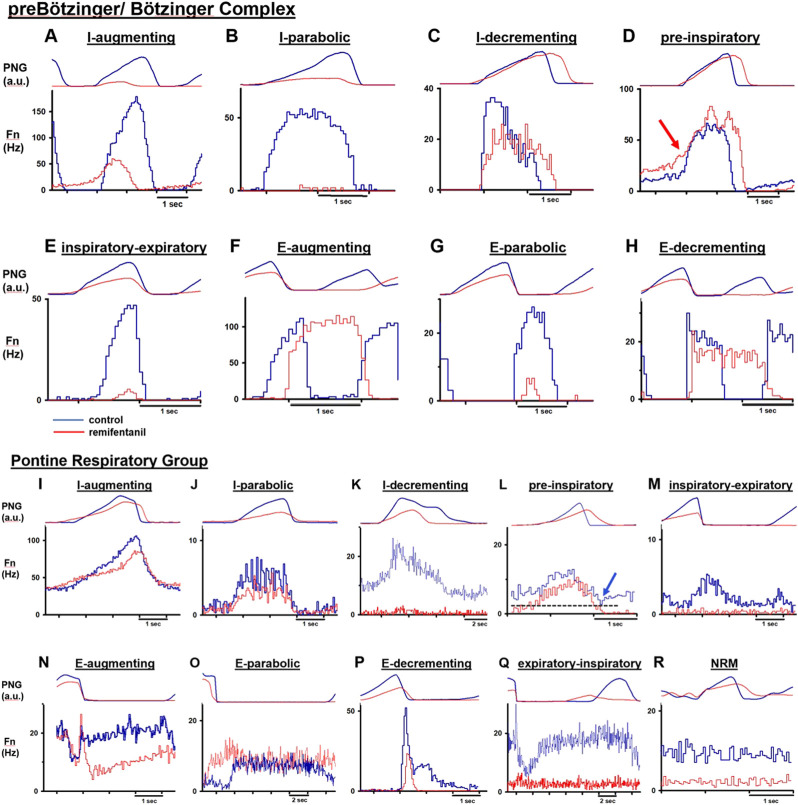
Cycle-triggered histograms before (blue) and during (red) remifentanil infusion allow characterization of neuron types by their discharge relative to the phrenic neurogram (PNG, arbitrary units). Neuronal discharge frequency (Fn) was often but not always depressed by remifentanil. **(A–H)** Neuron types recorded in the preBötzinger/ Bötzinger Complex. **(D)** red arrow: In 10/15 pre-inspiratory neurons, Fn was not decreased before start of inspiration. **(I–R)** Neuron types recorded in the Pontine Respiratory Group. **(L)** blue arrow: Characteristic for pre-inspiratory neurons was that Fn was lowest at the beginning of expiration and before start of the ramp pattern. Please note varying time scales. NRM: non respiratory-modulated.

Neurons were categorized by their main discharge phase as inspiratory (I) or expiratory (E) (Figure 2). We also analyzed several non-respiratory modulated neurons in the PRG. Since respiratory affiliation of these neurons was not clear, we limited analysis to a small sample of neurons with excellent signal quality located in close proximity to respiratory modulated neurons. Neurons were further grouped by discharge pattern, recognizing that separation into many subgroups decreases statistical power while summation of different neuron types into one group may confound subtype-specific remifentanil effects. Neuron subtypes matched those described in similar studies (Krolo et al., 2005; Smith et al., 2007; Rybak et al., 2008; Segers et al., 2008; Zuperku et al., 2019; Saunders et al., 2022), and especially studies of functional connections between PRG and preBötC/BötC (Rybak et al., 2008; Segers et al., 2008; Zuperku et al., 2019) to allow for functional interpretation of the results.

Discharge patterns were described as augmenting (aug) or decrementing (dec), however, we did not distinguish between, e.g., early- or late-inspiratory neurons since this attribution was somewhat arbitrary, and remifentanil often reduced neuronal discharge to part of the phase (e.g., Figures 2B, E, G). As no distinct post-inspiratory activity is observed in dogs, we counted potential post-inspiratory neurons as part of the E-dec population (e.g., Figures 2H, P) (Smith et al., 2007). Neurons without clear augmenting or decrementing discharge pattern were termed parabolic (para) (Figures 2B, E, J, O); this group included discharge patterns classified as plateau (Saunders et al., 2022), constant, or unspecified inspiratory or expiratory (Zuperku et al., 2019) in other publications. Inspiratory neurons showing a ramp-up of discharge activity during the expiratory phase were termed pre-inspiratory (pre-I) (Figures 2D, L). In PRG neurons, ramp-up could be difficult to distinguish from tonic activity prevalent in many pontine neurons. Neurons were only identified as pre-I when discharge activity was lowest at the beginning of expiration (Figure 2L, blue arrow). Phase-spanning neurons were classified as inspiratory-expiratory (IE) (Figures 2E, M), and expiratory-inspiratory (EI) (only pons, Figure 2Q). In the preBötC/BötC, IE neurons were difficult to distinguish from late-I neurons (Figure 2E), and only two neurons were included in that category.

### Remifentanil infusion protocol

**FIGURE 3:**
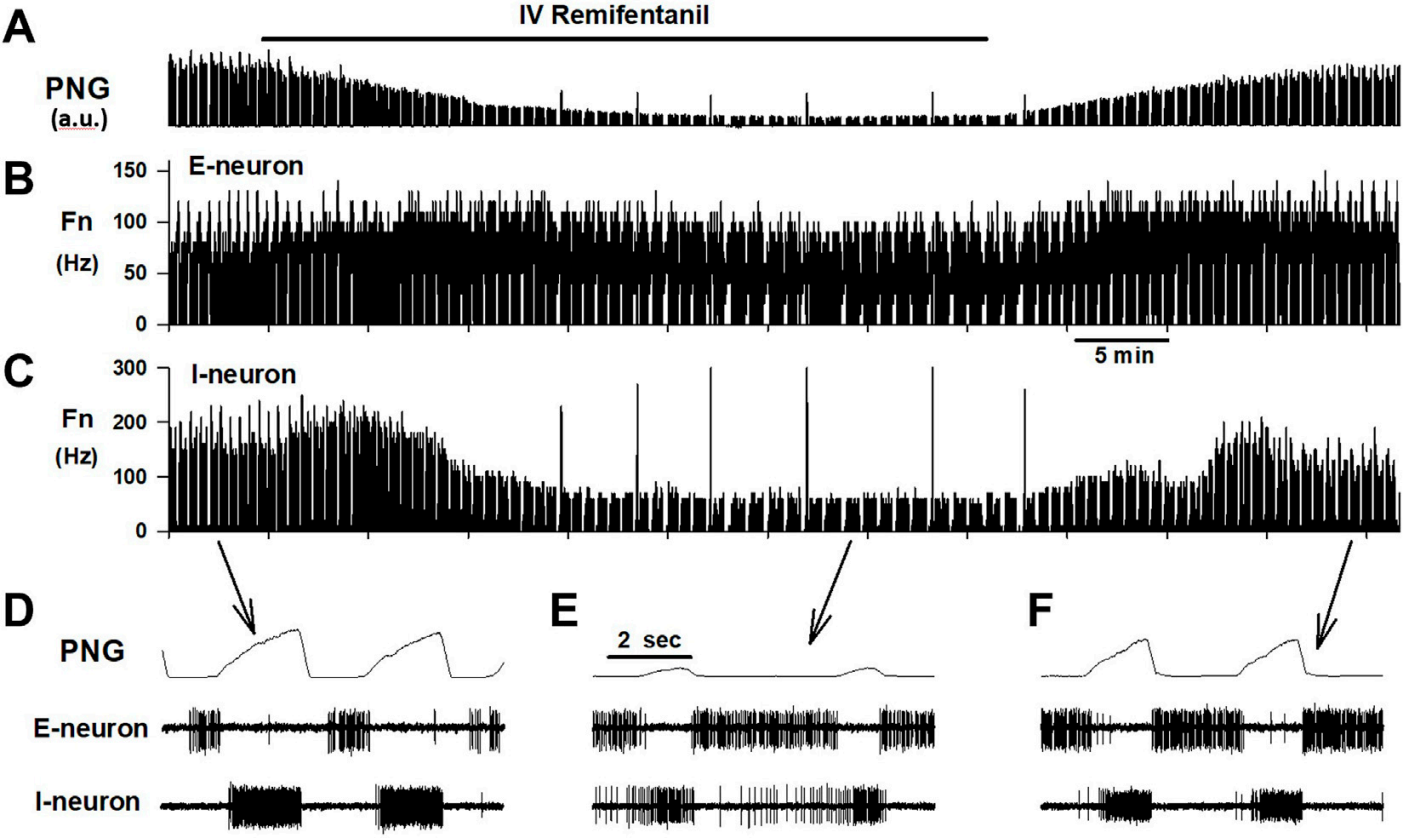
Example of an experimental protocol. Remifentanil was continuously infused for 40 min while PaO_2_ and PaCO_2_ were held constant with mechanical ventilation. **(A)** The phrenic neurogram (PNG, a.u.: arbitrary units) illustrates the decrease in respiratory rate and peak phrenic activity. Both recovered promptly after stopping the infusion. Concomitant extracellular recordings, here displayed as cycle-triggered histograms, show that remifentanil did not affect discharge frequency (Fn) of an expiratory neuron **(B)**, but severely depressed inspiratory neuronal discharge **(C)**. Time-expanded views of the PNG, and neuronal activity during **(D)** control, **(E)** maximal remifentanil effect, and **(F)** after recovery from remifentanil.

Remifentanil infusion was chosen due to its fast onset and short context-sensitive half-life of ∼4min, which is independent of the duration of the infusion (Burkle et al., 1996). Remifentanil infusions between 0.1–1 mcg/kg/min were used to reduce respiratory rate to 50%–60% of baseline (Michelsen et al., 1996; Michelsen and Hug, 1996) (Figure 3). Infusion rate was adjusted as necessary. Dosing regimen was similar to previous studies using naloxone injection during intravenous remifentanil infusion to compare local changes in neuronal discharge activity with previously described changes with nuclear injections (Mustapic et al., 2010; Prkic et al., 2012). During 30/110 remifentanil infusions, respiratory rate was *increased* even at the highest infusion rate and without obvious differences in animal state (temperature, CO_2_). An increase in respiratory rate with intravenous fentanyl has also been observed in goats (personal communication, Drs. Bert Forster and Matthew Hodges, MCW, 2019). In contrast to previous studies, we decided to include these data in the analysis. While the lack of a uniform endpoint for remifentanil dosing prohibited comparison of the magnitude of remifentanil effect on neuronal discharge between different subtypes, this allowed us to explore associations between changes in neuronal subtype Fn and concomitant changes in phase duration over a wider range, i.e., decreases as well as increases in phase duration.

Steady-state was accepted at least 10 min after the last change in remifentanil infusion rate. After capture of at least 20 respiratory cycles at steady-state for posthoc analysis, the remifentanil infusion was discontinued. Neuronal recordings were maintained until full recovery to control respiratory rate to ensure that decreases in Fn during remifentanil were due to the drug effect and not to loss of the neuronal recording.

### Data analysis

Cycle-triggered histograms were used to calculate peak discharge frequency (Fn) before and during remifentanil infusion. Similar to cats (Dick et al., 2008; Segers et al., 2008), most PRG neurons showed tonic discharge frequency with phasic modulation; peak discharge frequency was determined for the primary (more active) phase (I or E), and tonic activity was determined for the secondary (tonic) phase (E or I). “Phasic” activity was calculated as the difference between peak and tonic activity (see Supplementary Figure S1 for illustration). Since preBötzinger Complex neurons are mostly inactive between the primary phases, tonic activity was not calculated. Data were analyzed separately for the preBötC/BötC and PRG studies. For each study, Aim 1 was to determine the change in Fn with intravenous remifentanil for each neuron subtype. Aim 2 was to determine the relationship between % changes in Fn and concomitant % changes in TI, TE, or PPA. To avoid overestimation of the remifentanil effect on phase duration in runs with low baseline respiratory rate (i.e., long phase durations), we also calculated the input to inspiratory off-switch (derived from TI) and input to inspiratory on-switch (derived from TE) as previously described [Figure 2 in (Palkovic et al., 2020), Appendix 1 in (Palkovic et al., 2021)]. This calculation is based on the leaky integrator (LI) model where the sum of inputs to the integrator (σ) determines the time to reaching the threshold where the previous phase ends and the next phase starts [LI (*σ*) = σ*(1-exp^-t/τ^)]. For a threshold value of LI (*σ*) = 1, the sum of inputs can be calculated from the phase duration t, i.e., *σ* = 1/(1-exp^-t/τ^). For example, with *τ* = 1, a TE of 1s would compute to an input to inspiratory on-switch of 1/(1-exp^−1^) = 1.58, a TE of 2s would compute to an input of 1.16, and a TE of 10 s would compute to an input of 1.00005, i.e., barely above the (apneic) threshold of 1. This computation allows comparing changes in phase duration in proportion to the control phase duration and avoids overinterpretation of large changes in phase duration during very low respiratory rates. Neuron subtypes with less than 10 neurons were excluded from statistical analysis but are presented in the figures.

Data analysis required a model that allowed for multiple comparisons and for correlation analysis between remifentanil effects on neurons and phase timing while taking into account that often more than one run was performed per animal and more than one neuron of a certain discharge type was recorded during that run. This was in contrast to standard analysis of variance (ANOVA) testing where only one neuron is recorded per preparation and data points are thus independent. Data were analyzed using repeated measures generalized linear mixed models via PROC MIXED. This method was chosen because of its structural relationship to both repeated measures ANOVA and general linear models; generalized linear mixed models can perform a repeated measures analysis while controlling for separate trials within subjects via random effects. “Control” served as the baseline/reference for remifentanil. For Aim 1, the independent variable (fixed effect coefficient) was remifentanil, and the triad of animal, run, and neuron was controlled for via random effects. For Aim 2, the independent variable was the ratio of response within neuron, i.e., there was no need to control for the neuron effect. Since the % changes in TI, TE or PPA (dependent variables) were the same for all neurons of the same run, only the animal was controlled for via random effect. For PRG neurons, the ratio (Fn remifentanil/Fn control) was compared between phasic activity and tonic activity of the *same* neuron with the general linear mixed model (random effect: animal). A negative coefficient indicated that phasic activity was decreased more than tonic activity (which served as reference), and a positive coefficient that phasic activity was decreased less than tonic activity. A potential correlation between remifentanil-induced changes (ratios) in TI, TE, respiratory rate (RR), and PPA was tested with the general linear mixed model with animal controlled for via random effect. The coefficients of the fixed effects (Ce) are presented with 95% confidence intervals (C.I.), and inclusion of 0 in the confidence interval indicates no significant change. Larger fixed effect coefficients indicate greater effect size. We also provide *p*-values for the comparisons. Bonferroni corrections were performed for multiple comparisons. Considering the exploratory nature of this study, we consider associations “statistically significant” when *p* < 0.05 and/or confidence interval does not include 0. We also highlight possible associations when one or both of the ratio remifentanil/control and delta input to inspiratory on-/off-switch is statistically significant. Analysis was performed using SAS 9.4–14.3 (www.sas.com). Data were generally bell-shaped, and large enough for approximation to the normal per the Central Limit Theorem. Data descriptives presented are mean ± standard error.

## Results

### PreBötC/BötC study

**FIGURE 4:**
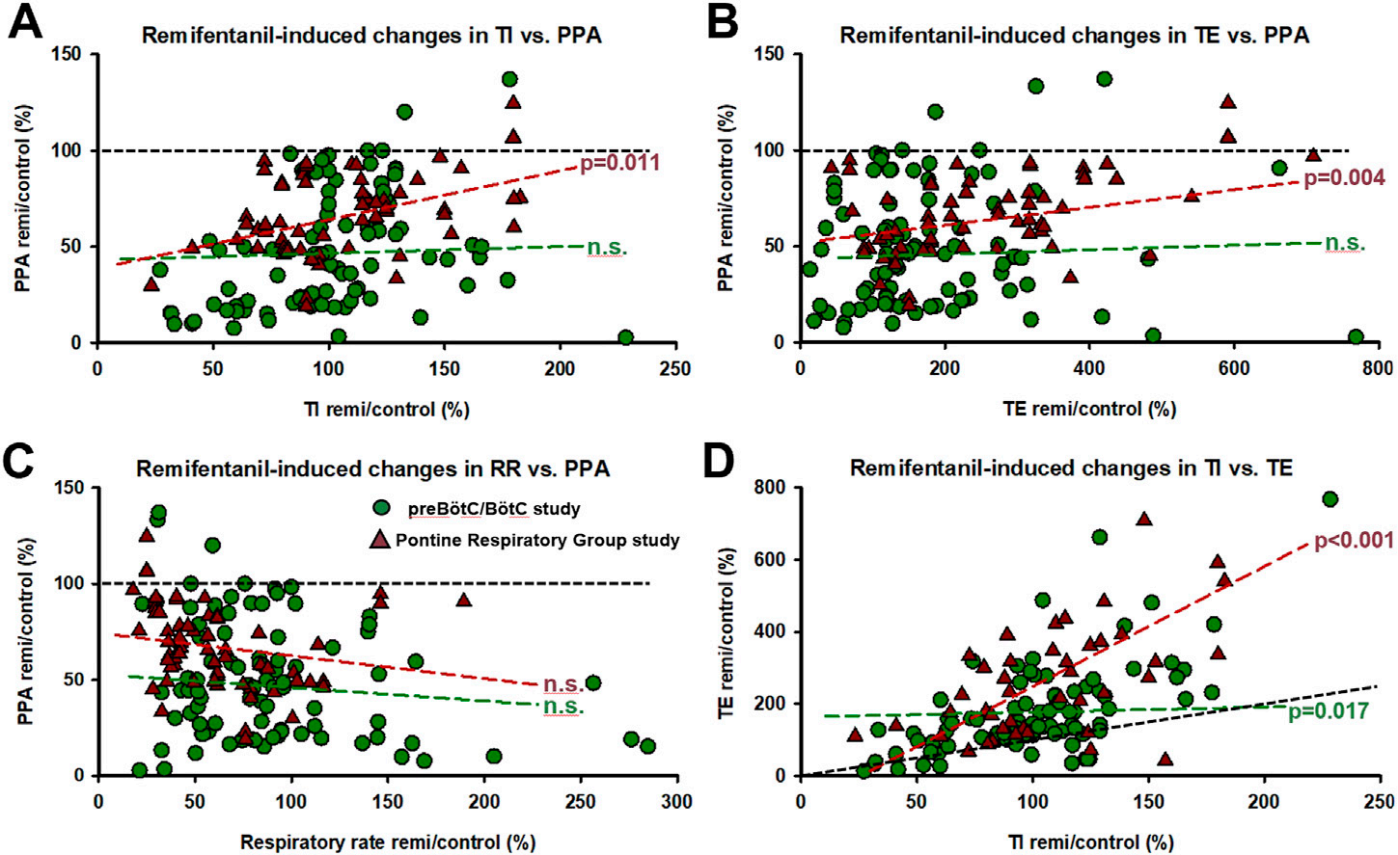
Corresponding remifentanil-induced changes for all experimental protocols in **(A)** inspiratory duration (TI) and peak phrenic activity (PPA), **(B)** expiratory duration (TE) and PPA, **(C)** respiratory rate (RR) and PPA, and **(D)** TI and TE. Generalized linear mixed model analysis (random factor: animal) showed no correlation between remifentanil-induced changes in TI, TE, RR, and PPA for the preBötzinger/Bötzinger Complex (preBötC/BötC) study (n.s.). In the PRG study, the correlation between changes in TI and PPA and TE and PPA was significant, however, PPA was depressed below baseline (black dotted line) even when TI was increased in the vast majority of cases. **(D)** Changes in TI and TE correlated significantly in both studies. In general, remifentanil increased TE more than TI (black dotted line, line of identity). Circles: preBötC/BötC study; triangles: pontine respiratory group study.

In the preBötC/BötC study, data were obtained from 36 animals, which yielded 213 neuronal recordings in 78 protocols. Baseline respiratory rate was 25 ± 2 breaths per minute (bpm). Remifentanil decreased respiratory rate in 56 protocols to 65% ± 3% of control and increased respiratory rate in 22 protocols to 191% ± 22%. There was no association between animal breed (mongrel, beagle) or sex and remifentanil effect on respiratory rate. In protocols with respiratory rate *decrease*, TI increased from 1.5 ± 0.1 s to 2.0 ± 0.4 s (137% ± 20%) and TE increased from 2.4 ± 0.4 s to 4.2 ± 0.5 s (227% ± 18%). In protocols with respiratory rate increase, TI decreased from 2.4 ± 0.2 s to 1.7 ± 0.3 s (65% ± 4%) and TE decreased from 3.5 ± 0.4 s to 2.0 ± 0.3 s (69% ± 5%). While remifentanil was more likely to cause a rate *increase* at low baseline respiratory rates (Supplementary Figure S2A), there was no clear relationship between remifentanil dose-rate and the direction and magnitude of the rate response (Supplementary Figure S2B). PPA decreased to 47% ± 4%, independent of the changes in TI (Figure 4A, Ce: 0.022, *p* = 0.258), TE (Figure 4B, Ce: 0.009, *p* = 0.710), and respiratory rate (Figure 4C, Ce: 0.074, *p* = 0.077). TI and TE generally changed in the same direction (Figure 4D, Ce: 0.107, *p* = 0.017).

**FIGURE 5:**
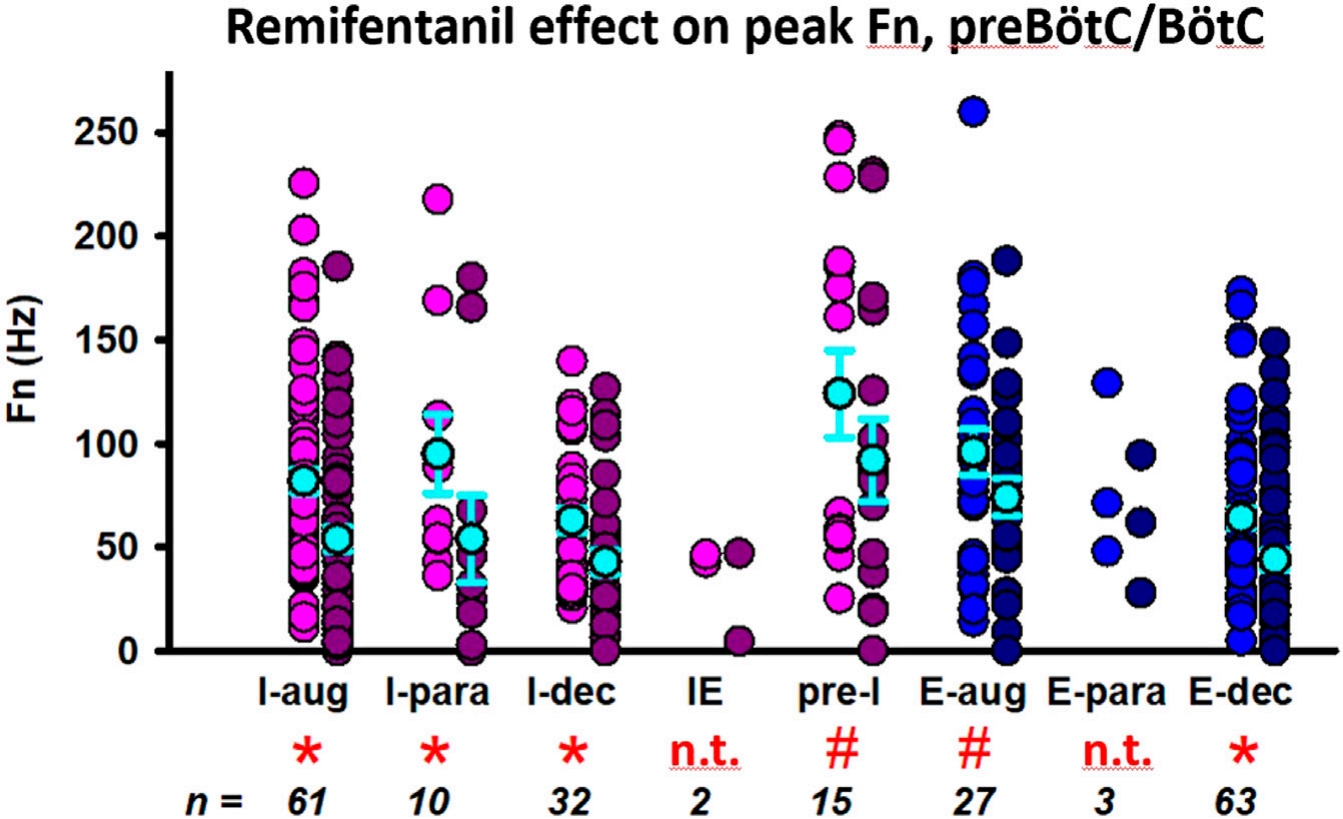
Remifentanil effect on preBötzinger/Bötzinger Complex neurons. Neuronal discharge frequencies (Fn) are pictured separately for each neuron type before (left) and during remifentanil infusion (right). *: *p* < 0.0083 (corrected for 6 comparisons); ^#^ *p* > 0.0083, however, the 95% C.I. did not include 0 suggesting a significant remifentanil effect. Neuron types with *n* < 10 were not included in the statistical analysis (n.t., not tested). Neurons were categorized by their discharge phase as inspiratory (I, pink) or expiratory (E, blue). Discharge patterns were described as augmenting (aug), parabolic (para), decrementing (dec), pre-inspiratory (pre-I), or inspiratory-expiratory (IE). The mean and standard errors for each data set are added in cyan for illustrative purposes; please note that the statistical analysis used a linear mixed model. For summary data including effect coefficient and confidence intervals, see Table 1.

Aim 1: Discharge frequency was decreased in I-aug neurons to 61% ± 6% (*n* = 61), in I-para neurons to 47% ± 12% (*n* = 10), in I-dec neurons to 66% ± 9% (*n* = 32), in pre-I neurons to 71% ± 12% (*n* = 15), in E-aug neurons to 88% ± 11% (*n* = 27), and in E-dec neurons to 72% ± 6% (*n* = 63) (Figure 5). There were too few IE (*n* = 2) and E-para (*n* = 3) neurons to allow statistical analysis. In a few neurons, remifentanil changed the discharge pattern, i.e., two I-aug neurons changed to I-dec, one I-para neuron changed to I-aug, one I-para neuron changed to I-dec, two I-dec neurons changed to I-aug. two E-aug neurons changed to E-dec, and one E-para neuron changed to E-dec. For a complete list of results including effect coefficients, C.I., and levels of significance, see Table 1.

**TABLE 1:** Effect of remifentanil on neuronal discharge frequency in the preBötzinger/Bötzinger Complex (preBötC/BötC) and Pontine Respiratory Group (PRG).

	Neuron type	n	Effect: remifentanil				Adjusted p	Depressed
			Coefficient	C.I. low	C.I. high	coefficient p		
preBötC/BötC	I-aug	61	−27.8287	−37.8668	−17.7906	<0.0001	*p* < 0.0083	*
	I-dec	32	−19.1531	−28.6716	−9.6346	0.0004		*
	I-para	10	−41.4831	−58.6127	−24.3535	0.0010		*
	pre-I	15	−31.4799	−59.3919	−3.5679	0.0442		#
	E-aug	27	−22.1111	−40.0294	−4.1928	0.0229		#
	E-dec	63	−20.1016	−27.8846	−12.3186	<0.0001		*
PRG	I-aug	144	−3.0424	−4.2347	−1.8501	<0.0001	*p* < 0.0056	*
	*I-aug tonic*		−1.2362	−1.6817	−0.7907	<0.0001		*
	I-dec	41	−1.1951	−3.7451	1.3549	0.3638		
	*I-dec tonic*		−1.8561	−2.7240	−0.9882	0.0001		*
	I-para	95	−3.3358	−4.5345	−2.1371	<0.0001		*
	*I-para tonic*		−1.4179	−1.8603	−0.9755	<0.0001		*
	pre-I	20	−2.3750	−5.4957	0.7457	0.1522		
	*pre-I tonic*		−0.9250	−2.8182	0.9682	0.3503		
	IE	28	−5.4286	−9.6144	−1.2428	0.0171		#
	*IE tonic*		−0.4942	−2.6937	1.7053	0.6633		
	E-aug	10	−1.1100	−4.7499	2.5299	0.5648		
	*E-aug tonic*		1.1500	−2.5385	4.8385	0.5563		
	E-dec	47	−9.5362	−12.2731	−6.7993	<0.0001		*
	*E-dec tonic*		−3.2064	−4.3107	−2.1021	<0.0001		*
	E-para	26	−3.0346	−6.2627	0.1935	0.0773		
	*E-para tonic*		0.1269	−2.4474	2.7012	0.9236		
	NRM	19	−4.1737	−8.0819	−0.2655	0.0508		#
	*NRM tonic*		−5.6475	−9.0058	−2.2892	0.0043		*

Neuron classification: I-augmenting (I-aug), I-parabolic (I-para), I-decrementing (I-dec), inspiratory-expiratory (IE), pre-inspiratory (pre-I), E-augmenting (E-aug), E-parabolic (E-para), E-decrementing (E-dec), non-respiratory modulated (NRM). For PRG neurons, changes in the phasic and tonic component of the discharge pattern were analyzed separately. Shown are the remifentanil effect (fixed effect coefficient) and 95% confidence interval (C.I.). The critical *p*-value was adjusted for the number of comparisons separately for the preBötC, PRG phasic activity, and PRG tonic activity. *: significant depression per *p*-value and C.I. does not include 0. #: *p*-value not significant, but C.I. does not include 0.

While the remifentanil-induced depression of pre-I neurons was relatively small (∼29%), the importance of these neurons for rhythmogenesis prompted us to perform additional analysis of the remifentanil effect on the expiratory phase ramp-like activity prior to inspiratory onset. We observed an increase in discharge frequency at I-onset, i.e., just before the rapid increase in pre-I neuronal discharge that commences with the start of the inspiratory phase, in 10/15 animals (Figure 2D), however, the pooled difference was not statistically significant [4.5 Hz (−2.1–15.1), mean (range), *p* = 0.05, Wilcoxon signed rank test]. However, remifentanil significantly decreased the average ramp-slope that was calculated as Fn (I-onset)/TE from 17.3 Hz/s (9.7–38.5) to 9.1 Hz/s (5.4–19.6, *p* = 0.005, Wilcoxon signed rank test, Supplementary Figure S3).

**FIGURE 6:**
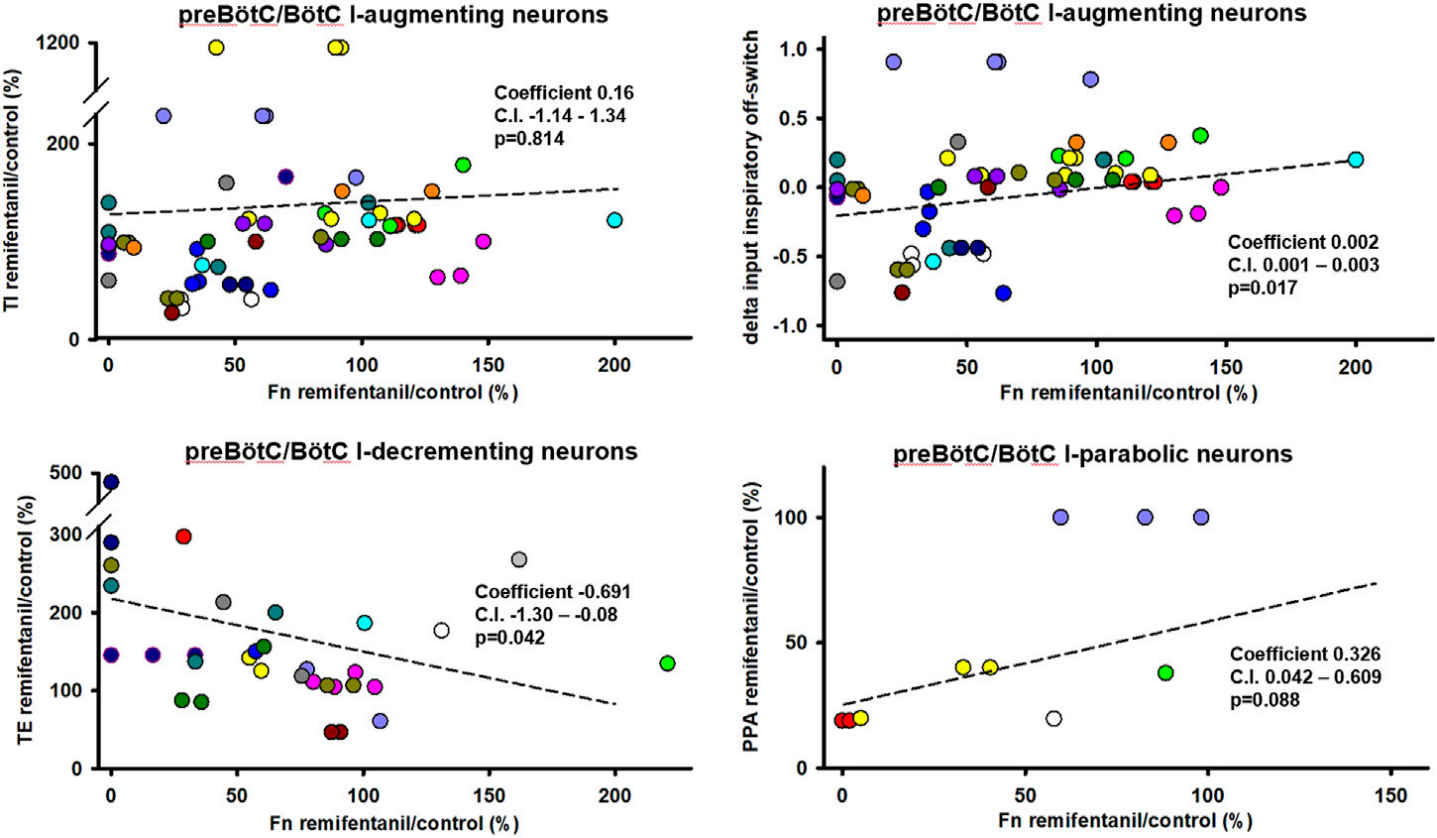
Statistical associations between the remifentanil-induced changes in neuronal discharge frequency [Fn remifentanil/control (%)] and changes in inspiratory duration (TI), expiratory duration (TE), or peak phrenic activity (PPA) were found for three neuron types in the preBötzinger/Bötzinger Complex. Colors indicate different animals, controlled for as random factor in the statistical analysis. For inspiratory (I)-augmenting neurons, correlations did not become significant for raw TI but only when changes in TI were “normalized” to control phase duration using the delta of the inputs to inspiratory off-switch. A positive coefficient indicates that the decrease in Fn was associated with a decrease in phase duration. A negative coefficient indicates that the decrease in Fn was associated with an *increase* in phase duration. Regression lines are interpolated based on the average intercept coefficient and ratio coefficient (slope) and do not reflect the linear mixed model analysis. For a summary of all tested effect coefficients and confidence intervals, see Table 2.

Aim 2: The remifentanil-induced decrease in I-aug discharge activity was associated with a decrease in TI (*p* = 0.017 for inputs to I-off-switch) (Figure 6). There was a possible relationship between remifentanil-induced decrease in I-para discharge activity and the decrease in PPA (*p* = 0.088, Ce: 0.3, C.I. 0.04–0.6, Figure 6). The decrease in I-dec activity was related to an *increase* in TE with a large Ce (−0.69, C.I. −1.30 to −0.08, *p* = 0.042). Interestingly, there was a large Ce for the association between decrease in E-dec activity and *increase* in TI, however, this was not significant (Ce −0.47, C.I. −1.23–0.29, *p* = 0.229). No other relationship was observed for preBötC/BötC neurons. For a list of all tested associations and results see Table 2.

**TABLE 2:** Association between remifentanil-induced respiratory discharge frequency (Fn) and inspiratory duration (TI), expiratory duration (TE), or peak phrenic activity (PPA) in the preBötzinger/Bötzinger Complex (preBötC/BötC) and Pontine Respiratory Group (PRG).

		Neuron type	n	Effect: change in Fn					Association
				Intercept coefficient	Ratio coefficient	C.I. low	C.I. high	Coefficient p	
preBötC/BötC	TI	I-aug	23	128.5	0.16	−1.14	1.45	0.814	
		*delta input I-aug*		−0.2	0.0021	0.0005	0.0037	0.0168	**#**
		I-dec	16	114.7	−0.13	−0.32	0.07	0.2138	
		I-para	5	63.0	0.28	−0.03	0.59	0.1546	
		pre-I	10	104.0	−0.02	−0.32	0.27	0.8889	
		Ea	14	78.0	0.08	−0.09	0.25	0.3931	
		E-dec	23	165.5	−0.47	−1.23	0.29	0.2288	
	TE	I-aug	23	211.3	0.16	−0.65	0.98	0.6954	
		I-dec	16	221.3	−0.69	−1.30	−0.08	0.0415	**#**
		I-para	5	96.6	0.23	−0.004	0.46	0.1268	
		pre-I	10	215.2	0.35	−1.21	1.91	0.6811	
		E-aug	14	142.3	0.01	−0.46	0.49	0.9544	
		E-dec	23	175.6	0.01	−0.61	0.63	0.9709	
	PPA	I-aug	23	44.1	0.02	−0.08	0.13	0.6384	
		I-dec	16	55.3	0.11	−0.10	0.32	0.9581	
		I-para	5	25.9	0.33	0.04	0.61	0.088	**#**
		pre-I	10	59.1	−0.08	−0.21	0.05	0.2928	
		E-aug	14	32.5	0.00	−0.06	0.07	0.9029	
		E-dec	23	47.5	0.06	−0.05	0.17	0.2813	
PRG	TI	I-aug	13	116.2	−0.12	−0.20	−0.05	0.0007	*
		I-dec	8	97.7	0.10	0.02	0.19	0.0254	**#**
		I-para	11	115.4	−0.13	−0.27	0.003	0.0593	
		*delta input I-para*		0.1	−0.0006	−0.0012	0.0000	0.0495	**#**
		pre-I	7	131.2	−0.15	−0.32	0.01	0.096	
		IE	7	93.3	0.13	−0.02	0.29	0.1134	
		E-aug	3	*did not converge*					
		E-dec	6	101.6	−0.03	−0.11	0.05	0.4571	
		E-para	8	95.4	−0.02	−0.02	−0.01	0.0007	*
		NRM	8	121.3	−0.08	−0.26	0.09	0.3755	
	TE	I-aug	13	261.2	0.02	−0.32	0.36	0.9134	
		I-dec	8	268.7	−0.08	−0.43	0.27	0.6568	
		I-para	11	213.6	0.37	−0.22	0.95	0.224	
		pre-I	7	446.3	−1.28	−3.09	0.53	0.1907	
		IE	7	169.2	1.33	−0.30	2.96	0.1254	
		E-aug	3	*did not converge*					
		E-dec	6	231.8	0.23	−0.44	0.90	0.5111	
		E-para	8	278.6	0.25	0.12	0.37	0.0011	*
		NRM	8	234.0	0.60	−0.20	1.40	0.1748	
	PPA	I-aug	13	60.3	0.06	0.02	0.10	0.0019	*
		I-dec	8	62.0	0.07	−0.04	0.19	0.2162	
		I-para	11	57.2	0.10	0.04	0.15	0.0007	*
		pre-I	7	69.1	0.03	−0.17	0.24	0.7622	
		IE	7	52.5	0.16	−0.0031	0.32	0.069	
		E-aug	3	*did not converge*					
		E-dec	6	61.3	0.02	−0.05	0.08	0.6467	
		E-para	8	75.5	−0.04	−0.10	0.01	0.1393	
		NRM	8	62.0	0.19	0.03	0.34	0.6467	**#**

Neuron classification: I-augmenting (I-aug), I-parabolic (I-para), I-decrementing (I-dec), inspiratory-expiratory (IE), pre-inspiratory (pre-I), E-augmenting (E-aug), E-parabolic (E-para), E-decrementing (E-dec), non-respiratory modulated (NRM). In two cases, the association between change in Fn and change in TI was not significant, however, there was an association when the input to inspiratory off-switch (derived from TI) was considered. Shown are the coefficient for the fixed effect (change in Fn) and 95% confidence interval (C.I.). The critical *p*-value was adjusted for the number of comparisons separately for the preBötC (*p* < 0.008, 6 comparisons) and PRG (*p* < 0.005, 10 comparisons). *: significant depression per *p*-value and C.I. does not include 0. #: *p*-value not significant, but C.I. does not include 0. Did not converge: data and model did not converge, no coefficient could be provided.

Additional analysis averaged the remifentanil-induced changes in TI, TE, and PPA for all runs for each individual neuron subtype. Overlap of the confidence intervals (C.I.) for the remifentanil effect (fixed effect coefficient) suggests that the remifentanil effect on phase timing was similar between the different neuron groups and that variation in the remifentanil effect on phase timing was likely not the reason for differences in remifentanil effect on the individual neuron subgroups (Table 3).

**TABLE 3:** Remifentanil-induced changes in TI, TE, and PPA averaged for all runs for each individual neuron subtype. Overlap of the confidence intervals (C.I.) for the remifentanil effect (fixed effect coefficient) suggests that the remifentanil effect on phase timing was similar between the different neuron groups and that variation in the remifentanil effect on phase timing was likely not the reason for differences in remifentanil effect on the individual neuron subgroups (Table 3).

preBötzinger/Bötzinger complex					Pontine respiratory group				
	Neuron type	Fixed effect coefficient	95% C.I.			Neuron type	Fixed effect coefficient	95% C.I.	
			Lower limit	Higher limit				Lower limit	Higher limit
TI	I-aug	0.8525	−0.2055	1.9105	TI	I-aug	0.0236	−0.0227	0.0699
	I_dec	0.0281	−0.1419	0.1982		I-dec	−0.3927	−0.6059	−0.1795
	I-para	−0.3400	−0.7481	0.0681		I-para	0.1316	0.0020	0.2612
	pre-I	−0.0400	−0.4884	0.4084		pre-I	0.1750	−0.0230	0.3730
	E-aug	−0.3889	−0.7194	−0.0584		IE	−0.1357	−0.3684	0.0970
	E-dec	0.3730	−0.2391	0.9851		E-aug	−0.1200	−0.7107	0.4707
						E-dec	−0.3404	−0.5081	−0.1727
						E-para	−0.1462	−0.4067	0.1143
						NRM	−0.3421	−0.6710	−0.0132
TE	I-aug	1.5000	0.9986	2.0014	TE	I-aug	6.7896	5.5673	8.0119
	I-dec	0.8375	0.2311	1.4439		I-dec	10.0659	6.7451	13.3867
	I-para	0.6100	−1.7204	2.9404		I-para	9.0063	6.8205	11.1921
	pre-I	2.0267	0.7507	3.3027		pre-I	9.5050	6.2193	12.7907
	E-aug	0.4259	−0.3122	1.1640		IE	5.9000	3.0423	8.7577
	E-dec	1.1333	0.4451	1.8215		E-aug	2.6600	−3.2341	8.5541
						E-dec	5.1596	2.9187	7.4005
						E-para	9.8000	7.0368	12.5632
						NRM	10.6526	6.4261	14.8791
PPA	I-aug	−54.8852	−63.6891	−46.0813	PPA	I-aug	−37.7361	−40.5226	−34.9496
	I-dec	−45.8750	−56.4537	−35.2963		I-dec	−23.1951	−28.9845	−17.4057
	I-para	−50.4000	−72.6176	−28.1824		I-para	−33.3895	−36.6811	−30.0979
	pre-I	−36.4000	−60.2557	−12.5443		pre-I	−27.6500	−35.2107	−20.0893
	E-aug	−64.5185	−72.3881	−56.6489		IE	−40.2857	−48.9789	−31.5925
	E-dec	−42.1429	−49.6127	−34.6731		E-aug	−35.2000	−49.4594	−20.9406
						E-dec	−43.7872	−47.3848	−40.1896
						E-para	−37.8462	−45.3610	−30.3314
						NRM	−22.2632	−29.5997	−14.9267

### PRG study

In the PRG study, data were obtained from 17 animals, which yielded 432 neuronal recordings in 42 protocols. Baseline respiratory rate was 10 ± 1 bpm. Remifentanil decreased respiratory rate in 34 protocols to 49% ± 4% and increased rate in 8 protocols to 122% ± 11%. Only beagles were used in this study. There was no association between sex and remifentanil effect on respiratory rate. In protocols with respiratory rate decrease, TI increased from 2.0 ± 0.3 to 2.1 ± 0.2 s (113% ± 11%) and TE increased from 6.4 ± 1.9 to 16.1 ± 4.3 s (300% ± 50%). In protocols with respiratory rate increase, TI decreased from 3.0 ± 0.6 to 2.4 ± 0.5 s (80% ± 15%) and TE decreased from 13.6 ± 3.9 to 10.6 ± 2.5 s (90% ± 11%). Again, remifentanil was more likely to cause a rate *increase* at low baseline respiratory rates (Supplementary Figure S2A), but there was no clear relationship between remifentanil dose-rate and the direction and magnitude of the rate response (Supplementary Figure S2B). PPA decreased independently of the change in respiratory rate to 68% ± 3%, and the change in PPA correlated with the change in TI (Figure 4A, Ce: 0.247, *p* = 0.011), and the change in TE (Figure 4B, Ce: 0.055, *p* = 0.004) but not respiratory rate (Figure 4C, Ce: −0.103, *p* = 0.203). In this dataset, too, the change in TI correlated with the change in TE (Figure 4D, Ce: 2.34, *p* < 0.001).

**FIGURE 7:**
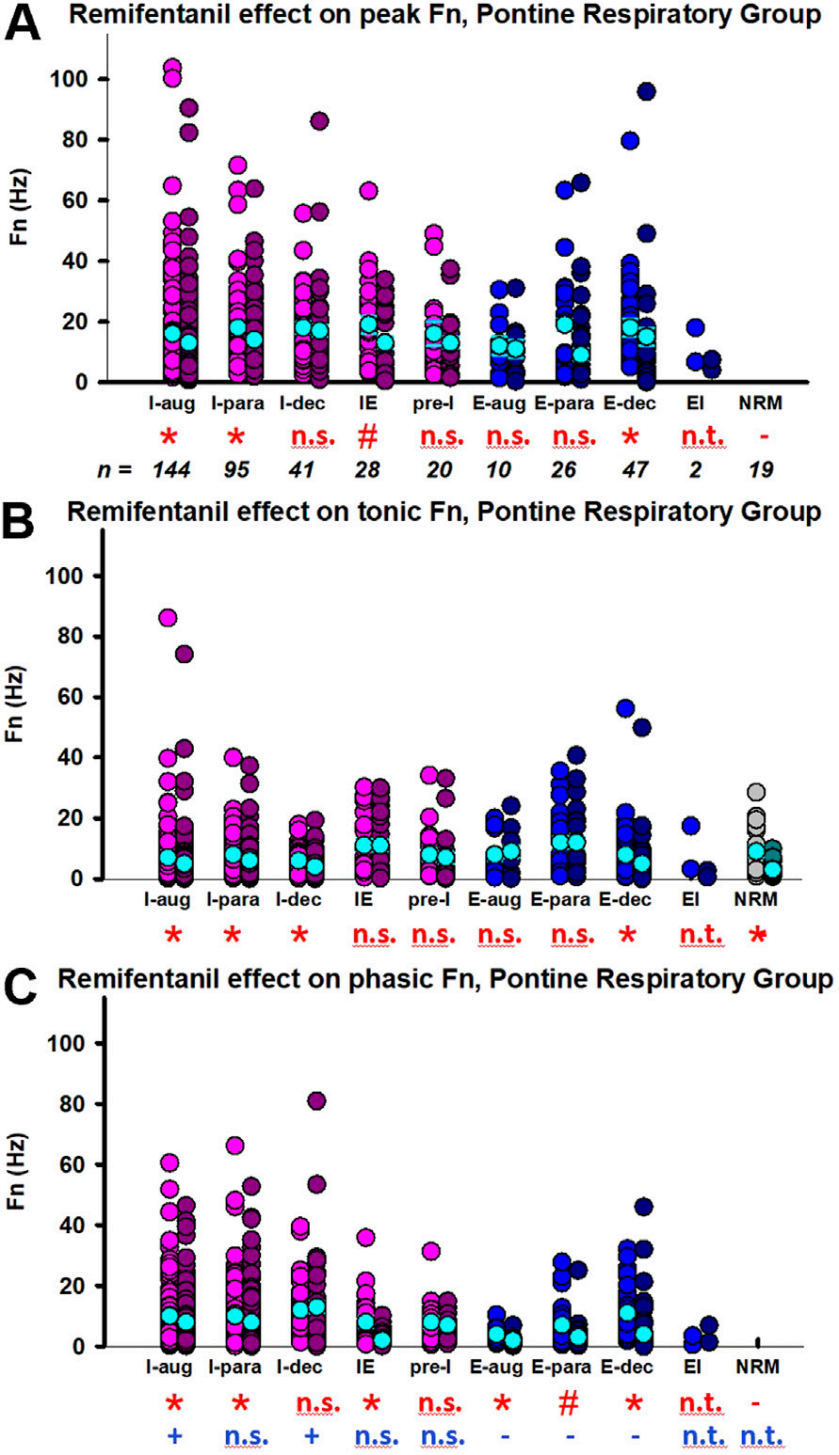
Remifentanil effect on pontine respiratory group (PRG) neurons. Neuronal discharge frequencies (Fn) are pictured separately for each neuron type before (left) and during remifentanil infusion (right). The discharge of most PRG neurons had a phasic and a tonic component. Pictured are the remifentanil effect on **(A)** peak Fn during the dominant phase, **(B)** Fn during the tonic (non-dominant) phase, and **(C)** the phasic modulation during the dominant phase (=peak Fn—tonic Fn). Red incidentals: remifentanil effect on individual neuron type; *: *p* < 0.0056 (corrected for 9 comparisons); #: *p* > 0.0056, however, the 95% C.I. did not include 0 suggesting a significant remifentanil effect. Blue incidentals: relationship between the remifentanil effect on tonic Fn **(B)** and phasic modulation **(C)**; +: phasic activity was decreased less than tonic activity; -: phasic activity was decreased more than tonic activity (linear mixed model, reference tonic). Neuron types with *n* < 10 were not included in the statistical analysis (n.t., not tested); n.s., not significant. Neurons were categorized by their main discharge phase as inspiratory (I, pink), expiratory (E, blue), or non-respiratory modulated (NRM, green). Discharge patterns were described as augmenting (aug), parabolic (para), decrementing (dec), pre-inspiratory (pre-I), inspiratory-expiratory (IE), and expiratory-inspiratory (EI). The mean and standard errors for each data set are added in cyan for illustrative purposes; please note that the statistical analysis used a linear mixed model. For summary data including effect coefficient and confidence intervals, see Table 1.

Aim 1: Remifentanil decreased *peak* discharge frequency of I-aug neurons to 86% ± 4% (*n* = 144), of I-para neurons to 80% ± 5% (*n* = 95), of IE neurons to 82% ± 11% (*n* = 28), of E-dec neurons to 40% ± 7% (*n* = 47), and of NRM neurons to 71% ± 11% (*n* = 19) (Figure 7A). Pre-I neuron (91% ± 11%, *n* = 20), E-aug neuron (96% ± 16%, *n* = 10), and E-para neuron activity (109% ± 10%, *n* = 26) was not decreased. Remifentanil decreased tonic discharge frequency of I-aug neurons to 83% ± 4%, of I-para neurons to 78% ± 5%, of I-dec neurons to 66% ± 8% (*n* = 41), of E-dec neurons to 49% ± 7%, and of NRM neurons to 62% ± 11% (Figure 7B). For a complete list of results including effect coefficients, C.I., and levels of significance, see Table 1. When comparing the phasic component of peak discharge frequency (Supplementary Figure S1) with tonic discharge, in I-aug and I-dec neurons, remifentanil decreased *phasic* activity less than tonic activity, while in E-aug, E-para, and E-dec neurons, phasic activity was decreased more than tonic activity (Figure 7C). However, due to the low neuronal discharge activity at baseline, absolute differences were small, and further analysis was limited to changes in *peak* discharge frequency. Remifentanil changed the discharge pattern of three I-dec neurons to I-aug, of one I-dec neuron to I-para, and of one I-para neuron to I-aug.

**FIGURE 8:**
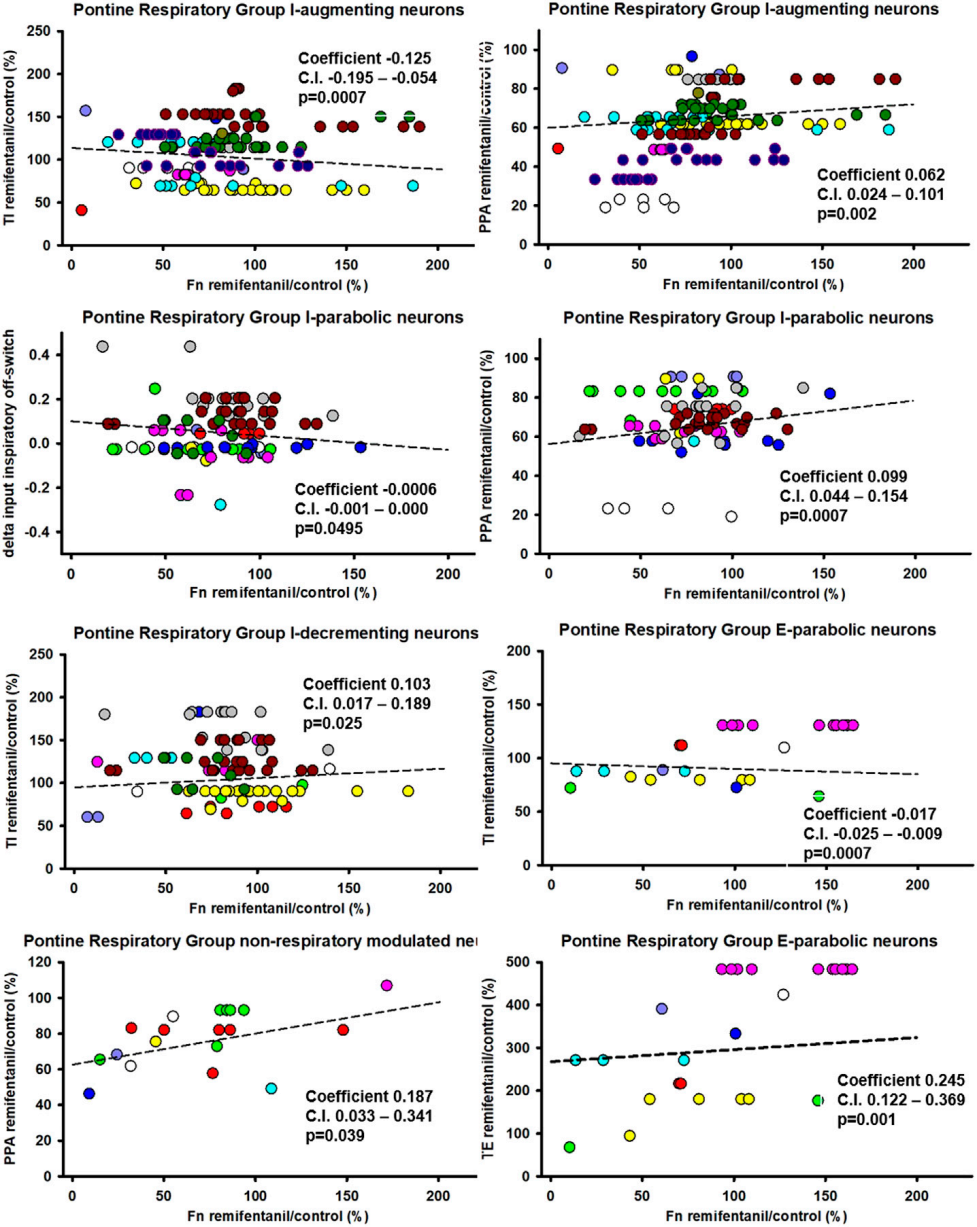
Statistical associations between the remifentanil-induced changes in neuronal discharge frequency [Fn remifentanil/control (%)] and changes in inspiratory duration (TI), expiratory duration (TE), or peak phrenic activity (PPA) were found for five neuron types in the Pontine Respiratory Group. Colors indicate different animals, controlled for as random factor in the statistical analysis. Positive coefficient indicates that the decrease in Fn was associated with a *decrease* in phase duration. Negative coefficient indicates that the decrease in Fn was associated with an *increase* in phase duration. *: there was no statistical significance for changes in TI with I-parabolic neurons, but statistical significance was observed for delta inputs to inspiratory off-switch (coefficient −0.0006, C.I. −0.0012-0, *p* = 0.05) suggesting an association. For a summary of all tested effect coefficients and confidence intervals, see Table 2.

Aim 2: The remifentanil-induced decreases in I-aug activity (*p* = 0.0007, *p* = 0.0003 for inputs to I-off-switch) and possibly I-para activity (*p* = 0.059, *p* = 0.05 for inputs to I-off-switch, Ce: −0.0006, C.I.: −0.0012-0, Figure 8) were associated with an *increase* in TI. In contrast, the decrease in I-dec activity was associated with a decrease in TI (*p* = 0.025, Ce: 0.10, C.I.: 0.02–0.19). Decreases in I-aug activity (*p* = 0.002, Figure 8) and I-para activity (*p* = 0.0007) were associated with a decrease in PPA. On average, E-para discharge activity was not significantly changed, however, there was a statistical association between changes in E-para activity and changes in phase duration such that a decrease in E-para activity was associated with an increase in TI (*p* = 0.0007) and a decrease in TE (*p* = 0.0011, Figure 8). Remifentanil-induced depression of *tonic* NRM neuron activity (*p* = 0.031, Ce: −0.22, C.I.: −0.39 to −0.05) was related to an *increase* in TI and a decrease in PPA (*p* = 0.039, Ce: 0.187, C.I.: 0.033–0.341). For a list of all tested associations and results see Table 2.

Additional analysis showed that the changes in TI, TE, and PPA were similar between the cohorts of the individual neuron subtypes (overlapping confidence intervals), suggesting that differences in the effect on neuronal discharge activity may not have resulted from a difference in remifentanil dosing (Table 3).

## Discussion

Our studies showed that remifentanil decreased the discharge frequency of nearly all types of preBöt/BötC neurons and many neuron types in the PRG. There was a strong link between remifentanil-induced changes in TI and TE that was maintained whether remifentanil increased or reduced the breathing frequency (Figure 4D). Remifentanil also decreased PPA, which was only weakly linked to the changes in phase duration (Figures 4A, B) and not to respiratory rate (Figure 4C). While remifentanil-induced increases in respiratory rate occurred only at lower baseline rates (Supplementary Figure S2A), there was no correlation between infusion rates and respiratory rate change (Supplementary Figure S2B).

**FIGURE 9:**
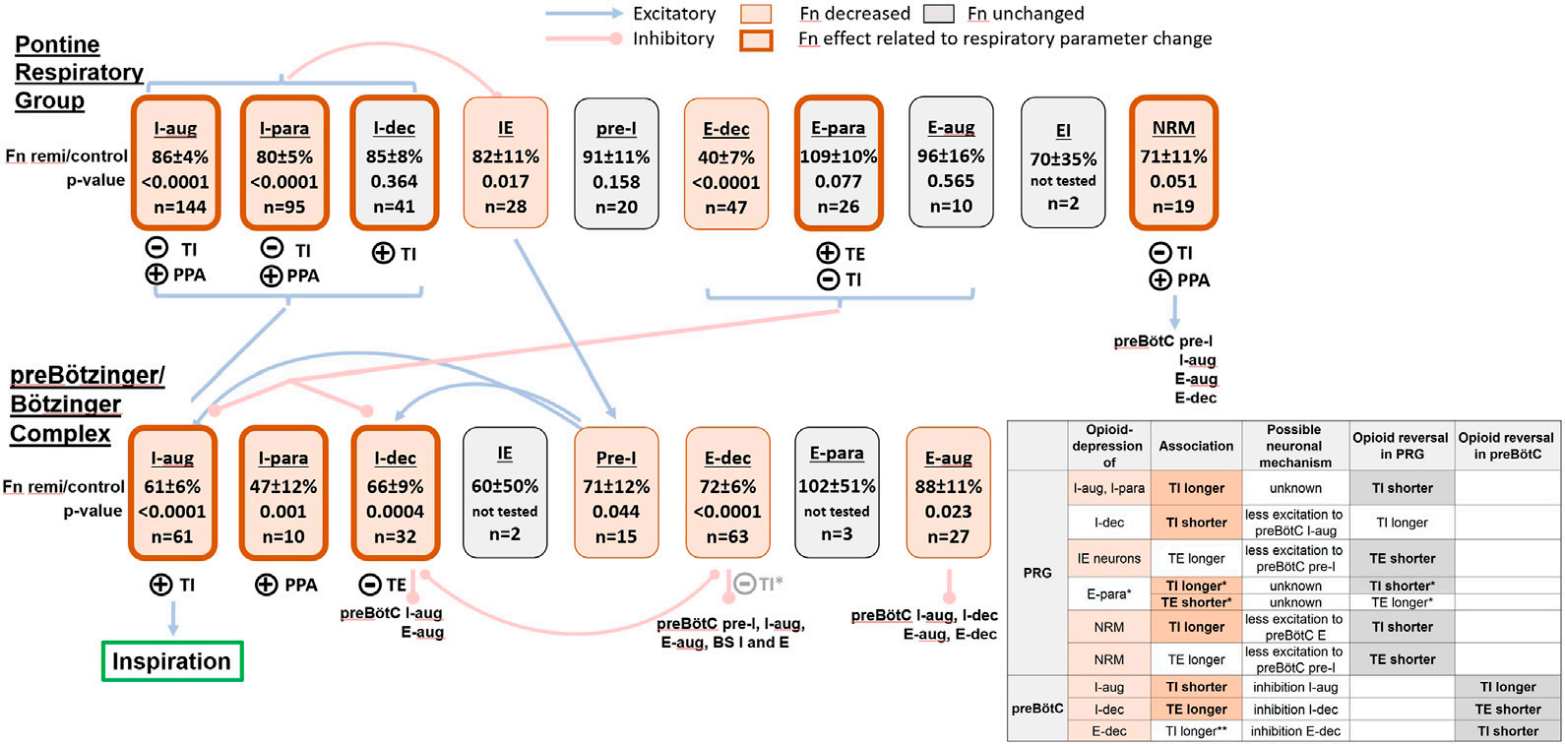
Hypothetic model of the remifentanil effect on the ponto-medullary respiratory pattern generator including neuronal connectivity described by Segers et al. (2008). For illustration, we list the average discharge frequency (Fn) during remifentanil relative to control for each neuron type and the number of recorded neurons in our studies. The level of significance was obtained from the linear mixed model. Neuron block shading indicates a significant remifentanil effect and encircling bold lines a significant relationship between changes in neuronal activity and phase-timing and/or peak phrenic activity (PPA); see legend at top of figure. Circled (+): the decrease in Fn was statistically associated with a decrease in inspiratory duration (TI), expiratory duration (TE), or PPA. Circled (-): the decrease in Fn was statistically associated with an *increase* in TI or TE. Neuron classification: I-augmenting (I-aug), I-parabolic (I-para), I-decrementing (I-dec), inspiratory-expiratory (IE), pre-inspiratory (pre-I), E-augmenting (E-aug), E-parabolic (E-para), E-decrementing (E-dec), expiratory-inspiratory (EI), non-respiratory modulated (NRM); BS: bulbospinal. Blue brackets: the referenced neuronal connectivity model did not distinguish between inspiratory and expiratory neuron subtypes in the Pontine Respiratory Group (PRG). Insert table: Possible neuronal mechanisms for the observed remifentanil effect. Column 1: Neuron subtypes in the preBötC and PRG that were significantly depressed are highlighted in salmon. Column 2: associated changes in TI or TE that were statistically significant are printed in bold. Other changes in phase duration are inferred from known synaptic connections (Segers 2008). Column 3: possible neuronal mechanism, based on known synaptic connections. We assume that the remifentanil effect of PRG neurons is mediated by their projections to preBötC/BötC neurons while the effect on preBötC/BötC neurons is direct inhibition. Column 4: change in phase duration that would be expected with opioid reversal in the PRG or preBötC/BötC (column 5). Changes that were observed experimentally with naloxone injection in our previous studies are highlighted in bold. *: Changes in PRG E-para activity was associated with changes in TI and TE; the lack of depression of this neuron type may result in a relatively unchanged TI and TE. **: large effect coefficient for the association of decreased preBötC/BötC E-dec activity and increase in TI, but not statistically significant. See Discussion for more detail.

The main goal of this study was an extensive statistical analysis to identify potential functional relationships between the effects of remifentanil on the activity of the various PRG and preBötC/BötC neuron subtypes and TI and TE. These statistical associations do not necessarily represent causal relationships, but they are useful in suggesting causal relationships, especially when used with other supporting evidence. We used the associations and a respiratory network model based on cross-correlation studies (Rybak et al., 2008; Segers et al., 2008) to propose potential neuronal mechanisms for the remifentanil-induced changes observed in our studies (Figure 9). Our analysis identified possible neuronal mechanisms for an opioid-induced increase in TI within the PRG, for the lack of such effect on TI within the preBötC/BötC, and for an increase in TE within the PRG and preBötC/BötC, which had been previously observed with localized naloxone injections (Mustapic et al., 2010; Prkic et al., 2012).

### Does opioid-induced depression of preBötC pre-I neurons increase expiratory phase duration?

Many authors have postulated that preBötC pre-I neurons are critical for promoting the inspiratory on-switch (Onimaru and Homma, 2006; Del Negro et al., 2018), and that a change in preBötC pre-I neuron activity was related to changes in TE (Zuperku et al., 2019). In the *in vitro* slice preparation, opioid application decreased the rate of preBötC “burstlets,” considered an expression of pre-I neuron activity, and also the number of population bursts (Sun et al., 2019). We also found that preBötC pre-I neuron activity was significantly decreased by remifentanil as was activity of PRG IE neurons, which provide excitatory inputs to pre-I neurons (Rybak et al., 2008; Segers et al., 2008); however, there was no statistical association with the change in TE (Table 2). This may be due to the relatively smaller depression (∼29%) of pre-I neurons compared to the other preBötC/BötC I neuron subtypes or the greater variance in opioid sensitivity that was described by Baertsch et al. (2021). It is also possible that peak discharge frequency is not the best indicator of neuronal excitability: In 10/15 pre-I neurons, the ramp activity during the expiratory phase immediately before I-onset was increased by remifentanil (Figure 2D, red arrow), although the pooled effect was not statistically significant (Supplementary Figure S3A). We interpret this as a decrease in neuronal excitability similar to Butera et al., Figure 1 (Butera 1999), where greater depolarizing inputs were necessary to elicit bursting behavior. It is possible that the increased ramp activity before I-onset was due to decreased inhibition from (depressed) E-dec neurons, however, considering the entire expiratory phase, remifentanil net-depressed the ramp-slope up to inspiratory-onset by ∼50% resulting in more time required to reach the threshold for burst generation and thus an increase in TE (Supplementary Figure S3B). This result was similar to observations in the mouse medullary horizontal slice preparation where DAMGO significantly depressed the inter-burst activity of mu-opioid receptor expressing preBötC neurons while the burst spike frequency was only modestly depressed (Baertsch et al., 2021). In addition, a recent canine study suggested that the post-quiescent burst period may be prolonged by inhibition from E-dec neurons that delay the ramp build-up of pre-I activity (Zuperku et al., 2021). The study used electrically-induced pulmonary stretch receptor inputs during the expiratory phase of test cycles to prolong TE while recording from E-dec neurons in the preBötC/BötC region. Pulmonary stretch receptor input increased E-dec and concurrently reduced pre-I neuron activity. Spike-to-spike cross-correlation analysis showed a significant decrease in probability of pre-I neuron discharge activity immediately following an E-dec neuron spike, indicating inhibition of the pre-I neuron by the E-dec neuron (Zuperku et al., 2021).

On the other hand, in the current study remifentanil decreased E-dec neuron activity, which would have reduced this effect. Thus, remifentanil may have directly depressed pre-I activity during the expiratory phase as well as possibly increased the threshold required for burst initiation (Figure 2D, Supplementary Figure S3A). Remifentanil likely also decreased presynaptic excitatory inputs to pre-I neurons (Baertsch et al., 2021); together these effects may have contributed more to the delay of inspiratory on-switch than the remifentanil effect on pre-I neuron peak discharge frequency. Our data also showed a statistical association between the decrease in preBötC/BötC I-dec neuron activity and increase in TE (Figure 9). Based on network synaptic connections, such association may be due to disinhibition of preBötC E-aug neurons from both I-dec and E-dec neurons that were depressed by remifentanil. E-aug neurons have been shown to inhibit pre-I neurons (Smith et al., 2013), and thus E-aug neuron disinhibition would increase pre-I neuron inhibition and, consequently, increase TE.

### Tonic and phasic activity of PRG neurons

In this study, the average peak discharge frequency of PRG neurons was less than 20 Hz across all neuron types and was markedly less than the peak discharge frequency of neurons in the preBötC/BötC (compare Figure 7 with Figure 5). Most PRG neurons showed a significant level of tonic discharge activity (median: 43% of peak Fn; all data pooled) with 80% of sampled neurons showing phasic modulation during the inspiratory phase. In contrast, there was essentially no tonic activity in the preBötC/BötC neurons. Several studies have suggested that the phasic activity of PRG neurons is the result of synaptic inputs from medullary respiratory neurons to tonically discharging neurons in the PRG (Cohen, 1958; Cohen, 1979; Dick et al., 1994). In decerebrate cats, respiratory related discharge of pontine neurons was completely eliminated by hemisection at the level of the acoustic tubercle combined with mid-sagittal section extending from the level of the acoustic tubercle to the inferior collicle (Cohen, 1958). Studies using antidromic activation or cross-correlation techniques have shown axonal projections from the medulla to the pons (Ezure, 2004) and from the pons to the medulla (Ezure and Tanaka, 2006; Zuperku et al., 2019). Retrograde staining showed afferent projections to the pneumotaxic center from many neurons in the ventral respiratory group (Kalia, 1977). Taken together, it appears that respiratory neurons in the ventral respiratory group are the main source of modulation of the PRG neurons. Our data show that both components of the PRG discharge pattern, phasic and tonic, are depressed by remifentanil (Figure 7). While statistically, in inspiratory neurons phasic activity was decreased less than tonic activity, and in expiratory neurons phasic activity was decreased more than tonic, absolute differences in Hz were small and changes went in the same direction. We propose that the two components are not independent of one another but are integral parts of the total discharge pattern.

Interestingly, changes in NRM neuron activity were associated with changes in TI (Figure 8), suggesting a role for NRM neurons in phase timing. This was in line with observations in rabbits where neurons in the functionally identified parabrachial and Kölliker-Fuse nuclei contained practically no neurons with phasic discharge pattern (Navarrete-Opazo et al., 2020, Discussion). In dogs, experiments that used AMPA microinjections into the tachypneic subregion of the PRG showed that the correlation between changes in PRG neuronal discharge frequency and respiratory rate was greater for NRM neurons compared with inspiratory modulated neurons (Zuperku et al., 2020). The r-value for the correlation exceeded 0.75 for all PRG neuron types. Thus, the tonic activity of PRG neurons may be a primary source of excitation to preBötC pre-I neurons (Zuperku et al., 2019). Several network models for rhythm and pattern generation assumed that the medullary network excitation was due to non-specific tonic inputs from PRG neurons (Smith et al., 2007; Smith et al., 2013). Based on canine studies that showed excitatory synaptic inputs from the parabrachial nucleus to medullary pre-I and other I-neurons and inhibitory inputs to E-neurons, both of which would increase respiratory rate (Zuperku et al., 2019), and opioid studies in dogs and rabbits showing that opioid effects in the PRG particularly affect expiratory duration (Prkic et al., 2012; Miller et al., 2017; Palkovic et al., 2021; Palkovic et al., 2022), we propose that opioids depress pontine NRM neurons, resulting in decreased excitation of preBötC pre-I neurons and an increase in TE.

### Neuronal mechanisms associated with the increase in TI during opioid exposure

Typically, systemic opioid administration causes a decrease in respiratory rate resulting from an increase in both, TI and TE (Figure 4 upper) (Lalley, 2006; Ren et al., 2009; Mustapic et al., 2010; Prkic et al., 2012; Varga et al., 2020; Palkovic et al., 2021; Palkovic et al., 2022). The increase could be completely reversed with naloxone injections into the pontine parabrachial region in decerebrate dogs (Prkic et al., 2012) and partially in rabbits (Palkovic et al., 2021; Palkovic et al., 2022). In vagotomized animals, lesions or pharmacological suppression of the Kölliker-Fuse nucleus caused apneusis (pathologically prolonged inspiration) (St John et al., 1971; Gautier and Bertrand, 1975; Feldman and Gautier, 1976; Navarrete-Opazo et al., 2020) whereas electrical stimulation within the PRG region could terminate inspiration, reset phase, and trigger inspiratory off-switch (Cohen, 1971). In the current dataset, the decreased activity of several pontine neuron types was statistically associated with an increase in TI. Since NRM pontine neurons have excitatory connections with several types of medullary neurons, a remifentanil-induced reduction in these pontine neurons would be consistent with an increase in TI (Figure 9 upper right). We are not aware of any neuronal connectivity that would tie the decrease in pontine inspiratory neuron activity to an increase in TI (Figure 9 upper left).

In the preBötC, at “analgesic” remifentanil concentrations causing 50% respiratory rate depression, naloxone injection did not affect TI in dogs (Mustapic et al., 2010) or rabbits (Palkovic et al., 2021), suggesting no direct effect of opioids on TI in the preBötC. Interestingly, at remifentanil concentrations that produced apnea naloxone microinjection into the preBötC caused an additional *increase in* TI in rabbits (Palkovic et al., 2021; Palkovic et al., 2022), suggesting that higher opioid concentrations decreased TI in the preBötC. These concentrations were not tested in dogs. In the current dataset, we observed a decrease in preBötC I-aug neuron activity that was associated with a decrease in TI (Figure 9). This matched reports by Takeda et al. (2001) who showed direct inhibition in 18/40 preBötC inspiratory neurons *in vitro*. In decerebrate cats, direct inhibition was observed in 6 bulbospinal and 3 propriobulbar I-aug neurons with intravenous fentanyl but only at doses ≥10 and 18 μg/kg, resp (Lalley, 2006). Intravenous morphine caused hyperpolarization of propriobulbar and bulbospinal neurons shortly after injection and a decrease in excitatory inputs later on (Haji et al., 2003).

We also found a large effect coefficient that suggested an association between the decrease in preBötC/BötC E-dec activity and an increase in TI. Several network models for rhythm generation have been based, to various degrees, on reciprocal inhibition between preBötC I-dec and E-dec neurons. In order to generate decaying discharge patterns, these neurons are assumed to contain a voltage-dependent conductance with slow dynamics that can produce a post-inhibitory rebound excitation. A simulation of this interaction shows that it can produce a sustained rhythm (Stuth et al., 2005). Other properties of this circuit show that a decrease in the tonic excitation, which is necessary for operation, results in an increase in rhythmic rate, while an increase in the mutual synaptic inhibition results in a decrease in rate. We do not know whether the decrease in preBötC/BötC I-dec and E-dec neuron activity that we observed, was due to reduced tonic excitation to these neurons or direct inhibition, which would be necessary to explain the association with increases in TI and TE (Figure 9). Most recent models are a hybrid containing pre-I neurons in conjunction with inspiratory and expiratory decrementing neurons (Richter and Smith, 2014). The role of the decrementing neurons is to create time-dependent discharge patterns (pattern generation vs. rhythm generation) and to control phase durations. In this scenario, a remifentanil-induced decrease in I-dec and E-dec neuron activity, through disinhibition of E-aug neurons, which inhibit pre-I neurons, may produce an increase in TE. The increase in TI may be due to the intrinsic linkage between TI and TE (Zuperku and Hopp, 1985), as well as the remifentanil effects on PRG NRM neurons. It is possible that direct depression of preBötC I-aug neurons contributes to complete inspiratory failure (apnea) at higher opioid concentrations.

### Mechanisms underlying the linkage between TI and TE

The quasi-linear relationship between changes in TI and TE (Figure 4D) is a well-known phenomenon observed under various conditions. When TI was shortened by pulmonary stretch receptor inputs during the inspiratory phase, TE was also shortened (e.g., Clark and Euler, 1972; Zuperku and Hopp, 1985). When TE was lengthened by pulmonary stretch receptor inputs during the expiratory phase, the following TI was increased (Zuperku and Hopp, 1985). Alternately, when TE was shortened by electrical stimulation in the tachypneic subregion of the parabrachial nucleus, the following TI was shortened (Zuperku et al., 2017). When neurons in the same subregion were inhibited by microinjections of DAMGO, TI and TE increased; this linkage phenomenon occurred without interventions in the preBötC region (Prkic et al., 2012). In vagotomized dogs, the phrenic ramp and burst generating circuitry appears to have dynamic limitations involving recovery time and post-inspiratory refractory time that became obvious when the preceding TE was progressively shortened by parabrachial nucleus subregion stimulation. As TE became shorter, the following TI became shorter and PPA decreased (Zuperku et al., 2017), suggesting that the burst generating mechanism required more time to fully develop depending on the preceding TE. In addition, there was a marked refractory period following the phrenic burst. Thus, the mechanism underlying the TE-TI linkage relationship may involve the intrinsic properties of phrenic regenerative burst and associated ramp generating properties. The TI-TE linkage, seen when TI was shortened by inspiratory-phase pulmonary stretch receptor inputs, may have been due to an effect on post phrenic-burst refractory period, possibly due to the magnitude of the post-inhibitory rebound in expiratory neurons and the burst after-hyperpolarization, which affects TE (Baertsch et al., 2018). These notions are supported by studies in neonatal mouse transverse brainstem slices that contained a subset of preBötC glutamatergic neurons (Dbx1+), which show pre-I activity and are thought to have enhanced burst-generating properties and be the drivers of the preBötC inspiratory rhythm (Baertsch et al., 2018). The magnitude of the post-burst after-hyperpolarizations of these neurons was related to the intensity of the burst, induced by current injections, as well as the refractory period, which lasted ∼3 s, which is similar to that following a phenic burst is dogs. In a Vgat-ChR2 slice, blue light during the preBötC burst-inspiratory phase shortened burst duration as well as the inter-burst “expiratory phase,” while the blue light stimulus applied during the inter-burst phase increased the phase duration and possibly the burst duration [Figure 4D in (Baertsch et al., 2018)]. These results mimic results in *in vivo* dogs where pulmonary stretch receptor inputs during the inspiratory phase shortened TI and subsequent TE, and during the expiratory phase, which increased TE and the following TI (Zuperku and Hopp, 1985). Thus, it is possible that preBötC inhibitory neurons, when presynaptically activated, mediate the phase timing responses (Baertsch et al., 2018). Interestingly, when Dbx1+ neurons were photonically activated during the inter-burst period, that period shortened similarly to parabrachial nucleus stimulation, which shortened TE (Zuperku et al., 2017). In a related canine study we showed that preBötC pre-I neurons received excitatory synaptic inputs from the parabrachial nucleus subregion (Zuperku et al., 2019), consistent with the Dbx1+ pacemaker neurons.

### Is opioid-induced depression of PPA due to depression of preBötC and PRG neurons?

Our results showed a statistical association between depression of PRG I-aug, I-para and NRM neuron and preBötC/BötC I-para neuron activity and a decrease in PPA, suggesting that both pontine and preBötC/BötC neurons contribute to PPA. However, in the same dog model microinjections of naloxone into the subregion of the medial parabrachial nucleus, which produces tachypnea (Zuperku et al., 2017), completely reversed remifentanil-induced bradypnea/apnea but did not reverse the decrease in PPA (Prkic et al., 2012). In a different set of animals, microinjections of DAMGO into the parabrachial nucleus reduced respiratory rate by 74% but did not change PPA. Application of DAMGO-saturated pledgets to the dorsal surface of the pons between the inferior colliculus and the superior cerebellar peduncle produced a marked bradypnea with a slight increase in PPA associated with the increase in TI (Prkic et al., 2012). These studies suggest that PRG neurons control phase timing but not PPA, and that the statistical associations we observed in the current study were not causal. A previous canine study where IV remifentanil infusion similarly increased TI and TE and decreased PPA showed that extensive bilateral naloxone injections into the preBötC area had no effect on phase timing or PPA. On the other hand, bilateral microinjections of DAMGO into the preBötC increased respiratory rate via decreases in TI and TE and caused a small depression of PPA (Mustapic et al., 2010). DAMGO injection onto individual inspiratory and expiratory premotor neurons in the caudal ventral respiratory group depressed neuronal discharge activity. However, the depression of neuronal discharge frequency during systemic remifentanil infusions similar to the current study could not be reversed with local naloxone injection onto individual premotor neurons (Stucke et al., 2008). It is possible that that study did not identify an opioid effect on neurotransmitter release from neurons presynaptic to the individual premotor neurons. In addition, opioid sensitivity of the phrenic motonucleus has not been studied *in vivo*, and it is possible that opioids directly depress the motoneurons as well as decrease the release of excitatory neurotransmitters from presynaptic neurons, which project to this area from the respiratory rhythm generator and areas of chemoreception. The role of the individual brainstem areas may be somewhat species-dependent: In cats, morphine applied to the ventral surface of the medulla produced a reduction in tidal volume with only a small change in respiratory rate (Hurle et al., 1985). In contrast, morphine applied to the dorsal pontine surface produced a marked decrease in respiratory rate and no changes in tidal volume (Hurle et al., 1985). In decerebrate rabbits, naloxone injection into the bilateral parabrachial and Kölliker-Fuse nuclei during IV remifentanil infusion partially reversed respiratory rate depression and also depression of PPA to a small degree (Palkovic et al., 2021). High remifentanil doses caused apnea in the phrenic neurogram after naloxone injections into the bilateral pontine respiratory group and preBötC although the respiratory rhythm continued in the vagal neurogram, suggesting direct opioid depression of the phrenic motonucleus (Palkovic et al., 2021). Naloxone injection into the caudal medullary raphe partially reversed PPA and respiratory rate depression (Palkovic et al., 2022).

In general, PPA was depressed in nearly all experiments whether TI was increased or decreased, suggesting that opioids depress PPA differently and possibly outside of the respiratory pattern generator (Palkovic et al., 2021). Opioid effects on tidal volume may originate from the “chemo-sensitive” ventral region of the medulla rather than from preBötC and PRG neurons, and the statistical associations observed in this study may have resulted from collinearity between PPA and inspiratory duration (Figure 4A).

### Methodological considerations

Hypothetical network model: One limitation of our model is that the projections and function of each recorded neuron were not identified, i.e., we were not able to distinguish between propriobulbar vs. premotor and excitatory vs. inhibitory neurons. Thus, it is hard to gauge whether the “average” neuron effect for each subtype is representative of the individual neuron’s function and projections. Also, lack of homogeneity and low numbers for individual subtypes may have resulted in a lack of power to determine additional relationships between changes in neuronal activity and respiratory parameters. As noted above, statistical associations do not necessarily imply a causal mechanism but may be correlational in nature. Phasic modulation in the PRG presents a reflection of preBötC activity (Dutschmann and Dick, 2012) and may characterize the neuronal afferents but not their function.

Extracellular recordings cannot show whether neuronal discharge frequency was depressed due to decreased excitatory inputs, increased inhibitory inputs, or direct neuronal inhibition. Levitt et al. showed that ∼60% of Kölliker-Fuse neurons were directly inhibited by mu-opioid receptor agonists (Levitt et al., 2015; Varga et al., 2020), however, the lack of phasic activity in the slice preparation did not allow identification of the neuron type. Baertsch et al. reported that between 40% and 60% of the recorded preBötC inspiratory, pre-I, expiratory and NRM neurons expressed the Oprm gene (Baertsch et al., 2021).

In general, our model focused on a loss of *excitatory* inputs from the PRG to the preBötC/BötC. Such a view may be too simplistic considering that in a neonatal pons-medulla-spinal cord preparation, the bath-applied mu-opioid receptor agonist slowed burst frequency (“respiratory rate”) in the absence of the pons but increased rate when the pons was attached, suggesting facilitation of the medullary pattern generator by inhibition of the PRG (Tanabe et al., 2005). Zuperku et al. (2019) found in decerebrate dogs that electrical stimulation in the PRG caused a change in inputs in more than 50% of recorded medullary respiratory neurons; inspiratory and pre-I neurons received mostly excitatory inputs while expiratory neurons received substantially more inhibitory inputs.

Remifentanil concentration: The current studies were designed to determine the remifentanil effect on individual neurons at dose-rates similar to previous studies, i.e., at ∼50% respiratory rate depression (Mustapic et al., 2010; Prkic et al., 2012). In contrast to those studies, the present studies included runs in the analysis where remifentanil increased respiratory rate. This made it impossible to compare effects at the same TI and TE, however, it allowed us to explore a wider range of *changes* in TI and TE and associated changes in neuronal discharge frequency. The range of TI and TE changes with remifentanil infusion overlapped between most neuron subgroups (Table 3) suggesting that the remifentanil effect was on average similar between groups, however, we limited our interpretation to remifentanil-induced *change*s in Fn and corresponding changes in TI, TE, and PPA and did not compare the magnitude of depression between neuron subtypes. Increases in respiratory rate with intravenous fentanyl were routinely observed in a subset of awake goats (personal communication, Drs. Bert Forster and Matthew Hodges, MCW, 2019), and a biphasic respiratory rate response was observed with multiple doses of intravenous morphine in cats (Haji et al., 2003).

Baseline respiratory rate was on average lower in the PRG studies, possibly due to loss of cerebellar inputs or a general effect of the more extensive surgical preparation. Still, animals tolerated multiple remifentanil runs with recovery to their individual baseline respiratory rates, and there was no qualitative difference in remifentanil effect on phase duration between both studies (Figure 4). In 32 animals, we performed more than one protocol run (Supplementary Figure S2C). In most animals, remifentanil changed respiratory rate in the same direction, i.e., always caused an increase or decrease, however, in ten animals, remifentanil caused an increase and decrease in consecutive runs. There was no clear pattern to the magnitude of changes in consecutive runs and no suggestion of tolerance to the remifentanil effect. To avoid possible bias from different baseline respiratory rates, we did not compare remifentanil effects between the two brainstem sites but analyzed both datasets separately.

Neuron locations: We used recording locations identified in previous studies with injections of glutamate agonists and opioid agonists and antagonists (Krolo et al., 2005; Mustapic et al., 2010; Prkic et al., 2012). Based on the rostral location, we assumed that our PRG recordings were located in the parabrachial nucleus. However, the opioid-induced significant depression of inspiratory neurons and little depression of E-aug neurons was similar to a recent study in the Kölliker-Fuse nucleus in the *in-situ* rat preparation (Saunders et al., 2022). Neuron discharge types and a discharge pattern consisting of tonic activity with phasic modulation were similar to PRG recordings in cats (Rybak et al., 2008; Segers et al., 2008). Our “preBötC/BötC” area recordings likely included Bötzinger complex expiratory neurons, and bulbospinal neurons of the rostral ventral respiratory group in keeping with the expanded view of the medullary respiratory control network proposed by Baertsch et al. (2019). Our sample likely did not include many I-aug bulbospinal neurons since we would have expected a stronger relationship between neuronal depression and change in PPA.

Limitations of the animal model: Compared to awake animals, our acute decerebrate dog model has a reduced overall level of “respiratory drive.” Decerebration eliminates forebrain inputs (Hugelin and Geiger, 1986), reduces the hypercapnic ventilatory response (Mitchell, 1990), and additionally eliminates “awake drive,” which independently increases respiratory output (Montandon and Horner, 2019). We found that decerebration increased the apneic threshold compared to halothane anesthesia and decreased respiratory rate by 35% compared to awake, quietly breathing dogs (Stuth et al., 2000). Respiratory reflexes, neuronal discharge types and discharge frequency were similar to prior studies in thiopental, halothane, or isoflurane-anesthetized dogs (Zuperku and Hopp, 1985; Dogas et al., 1995; Krolo et al., 1999). To increase respiratory drive, we performed experiments at moderate hypercapnia (PCO_2_ ∼50 mmHg). Bilateral vagotomy was performed to achieve a regular respiratory pattern in these mechanically ventilated animals; this removed inhibitory inputs to the preBötC (Zuperku et al., 2021), resulting in a slower respiratory rate with increased PPA. The main difference to studying opioid effects in awake animals was that PO_2_ and PCO_2_ in our preparation were fixed with controlled ventilation. In contrast to freely behaving animals, animals in our experiments did not develop hypoxia and hypercapnia with increasing remifentanil concentrations, which would have alleviated the opioid-induced respiratory depression. We used hyperoxia to minimize inputs from peripheral chemoreceptors; this likely obscured remifentanil inhibition of the carotid bodies and nucleus tractus solitarii. Since it is not clear which neurons would have received additional excitatory or inhibitory inputs from hypoxia and hypercapnia, this may have led us to over- or underestimate the importance of certain neuronal mechanisms for the observed respiratory changes.

In conclusion, our data suggest mechanisms for how remifentanil decreased activity of preBötC/BötC pre-inspiratory, inspiratory decrementing and expiratory decrementing neurons and increased expiratory duration. We propose that a remifentanil-induced decrease in the ramp-up of preBötC/BötC pre-I neuron activity during the expiratory phase delays reaching the threshold for inspiratory burst initiation and thus increases TE. The decrease in excitatory inputs from PRG neurons likely contributes to this effect. Overall, our results support that pontine neurons play a key role in the control of phase timing and that intrinsic mechanisms within the preBötC/BötC burst and pattern generation circuits play a role in the linkage of TE and TI. While the decrease in activity of pontine and preBötC/BötC inspiratory neurons was associated with a decrease in peak phrenic activity, PPA was depressed even when neuronal discharge activity was increased, suggesting that the remifentanil effect on PPA is mediated mainly outside of the preBötC/BötC and PRG. Additional studies must clarify whether the observed changes in neuronal activity are due to direct opioid inhibition or decreased excitatory drive and thus whether these neurons may be useful targets for respiratory stimulating compounds.

## Data Availability

The raw data supporting the conclusion of this article will be made available by the authors, without undue reservation.

## Supplementary material

SUPPLEMENTARY FIGURE S1 Analysis model for neuronal discharge frequency (Fn) for neurons displaying tonic discharge pattern with phasic modulation in the pontine respiratory group. Peak discharge frequency (Fpeak) was determined for the primary (more active) phase (I or E), and tonic activity (Ftonic) was determined for the secondary (tonic) phase (E or I). “Phasic” activity (Fphasic) was calculated as the difference between peak and tonic activity. Inspiratory phase was determined from the phrenic neurogram (PNG).

SUPPLEMENTARY FIGURE S2
**(A)** Percent of animals that showed an increase versus decrease in respiratory rate at the “final” remifentanil infusion rate where neuronal activity was measured. Animals are grouped by baseline respiratory rate and presented separately for preBötzinger/Bötzinger Complex and Pontine Respiratory Group studies. In general, remifentanil increased respiratory rate only at lower baseline rates. **(B)** Changes in respiratory rate did not correlate with the remifentanil infusion rate in the preBötzinger/Bötzinger Complex (blue) and Pontine Respiratory Group (red) study. **(C)** Percent changes in respiratory rate with remifentanil infusion in consecutive runs in one animal. Data from animals where only one run was performed are not included.

SUPPLEMENTARY FIGURE S3 Additional analysis of the expiratory-phase ramp-up in discharge frequency of preBötzinger/Bötzinger Complex pre-inspiratory neurons. **(A)** Neuronal discharge frequency (Fn) at the end of the expiratory ramp, i.e., immediately before inspiratory onset was unchanged (*n* = 15). On the other hand, **(B)** the slope of the expiratory ramp, i.e., Fn before inspiratory onset/ expiratory duration (TE) was significantly decreased with remifentanil, suggesting that the delay until the burst threshold was reached may have contributed to the increase in TE. Wilcoxon signed rank test, *n* = 15.
