# Supplementary figures and images for: Changes in pontine and preBötzinger/Bötzinger complex neuronal activity during remifentanil-induced respiratory depression in decerebrate dogs
Source: Front Physiol. 2023 Jun 8;14:1156076. doi: 10.3389/fphys.2023.1156076 (PMC10285059; doi:10.3389/fphys.2023.1156076)

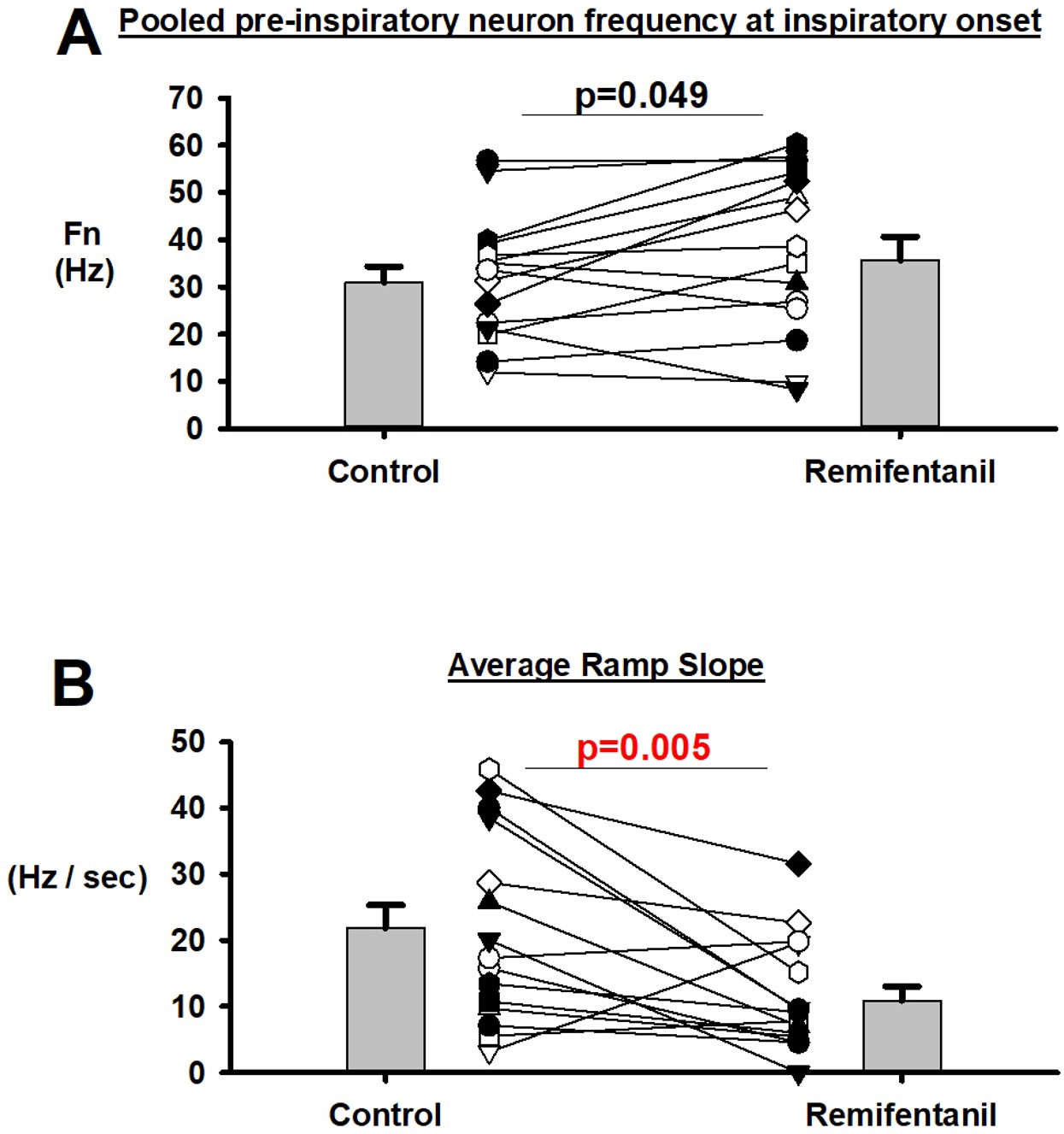

Supplement: Supplementary file 1 [file Image3.jpg]

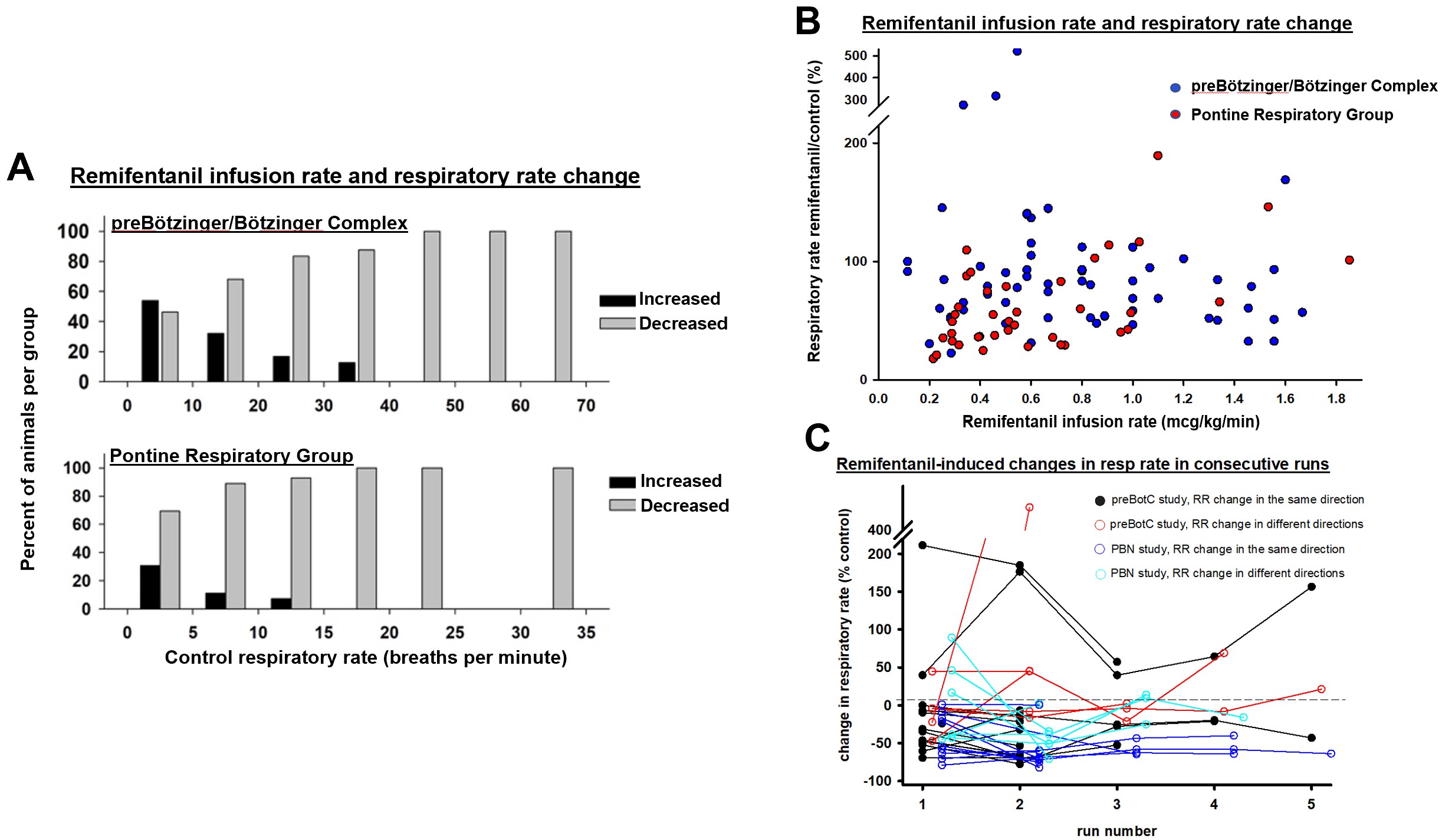

Supplement: Supplementary file 2 [file Image2.jpg]

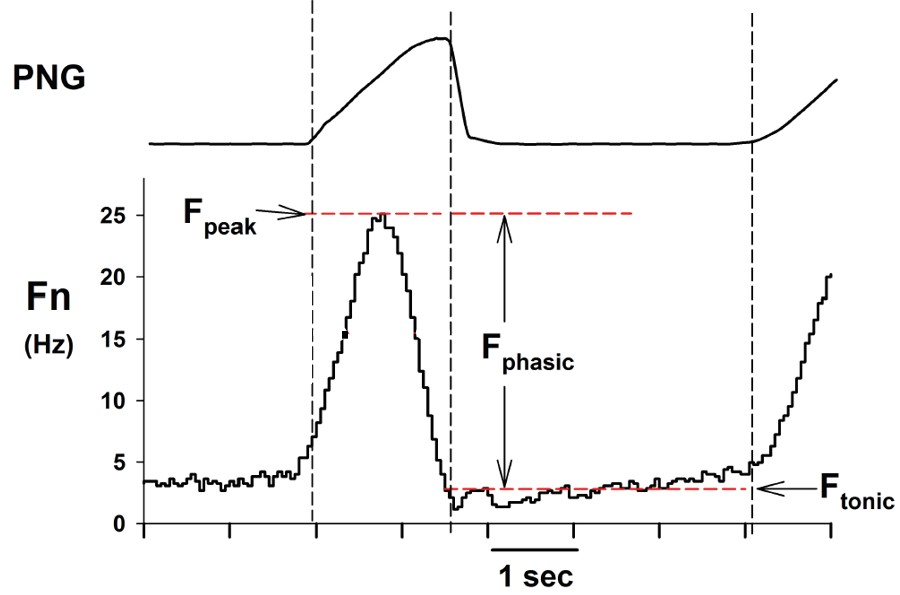

Supplement: Supplementary file 3 [file Image1.jpg]
